# Endometrial DNA methylation signatures during the time of breeding in relation to the pregnancy outcome in postpartum dairy cows fed a control diet or supplemented with rumen-protected methionine

**DOI:** 10.3389/fgene.2023.1267053

**Published:** 2024-01-24

**Authors:** Dessie Salilew-Wondim, Ernst Tholen, Eva Held-Hoelker, Karl Shellander, Carina Blaschka, Marc Drillich, Michael Iwersen, David Suess, Samuel Gebremedhn, Dawit Tesfaye, Claudia Parys, Ariane Helmbrecht, Jessie Guyader, Dennis Miskel, Nares Trakooljul, Klaus Wimmers, Michael Hoelker

**Affiliations:** ^1^ Department of Animal Science, Biotechnology and Reproduction of Farm Animals, University of Göttingen, Göttingen, Germany; ^2^ Institute of Animal Sciences, Animal Breeding, University of Bonn, Bonn, Germany; ^3^ Clinical Unit for Herd Health Management, University Clinic for Ruminants, Department for Farm Animals and Veterinary Public Health, University of Veterinary Medicine Vienna, Vienna, Austria; ^4^ Department of Biomedical Sciences, Animal Reproduction and Biotechnology Laboratory, Colorado State University, Fort Collins, CO, United States; ^5^ Evonik Operations GmbH, Hanau, Germany; ^6^ Research Institute for Farm Animal Biology (FBN), Dummerstorf, Germany

**Keywords:** methylation, pregnancy, endometrium, methionine, postpartum

## Abstract

Post calving metabolic stress reduces the fertility of high producing dairy cows possibly by altering the expression of genes in the maternal environment via epigenetic modifications. Therefore, this study was conducted to identify endometrial DNA methylation marks that can be associated with pregnancy outcomes in postpartum cows at the time of breeding. For this, twelve days post-calving, cows were either offered a control diet or supplemented daily with rumen-protected methionine. Cows showing heat 50–64 days postpartum were artificially inseminated. Endometrial cytobrush samples were collected 4–8 h after artificial insemination and classified based on the pregnancy out comes as those derived from cows that resulted in pregnancy or resulted in no pregnancy. The DNAs isolated from endometrial samples were then subject to reduced representative bisulfite sequencing for DNA methylation analysis. Results showed that in the control diet group, 1,958 differentially methylated CpG sites (DMCGs) were identified between cows that resulted in pregnancy and those that resulted in no pregnancy of which 890 DMCGs were located on chr 27: 6217254–6225600 bp. A total of 537 DMCGs were overlapped with 313 annotated genes that were involved in various pathways including signal transduction, signalling by GPCR, aldosterone synthesis and secretion. Likewise, in methionine supplemented group, 3,430 CpG sites were differentially methylated between the two cow groups of which 18.7% were located on Chr27: 6217254–6225600 bp. A total of 1,781 DMCGS were overlapped with 890 genes which involved in developmental and signalling related pathways including WNT-signalling, focal adhesion and ECM receptor interaction. Interestingly, 149 genes involved in signal transduction, axon guidance and non-integrin membrane-ECM interactions were differentially methylated between the two cow groups irrespective of their feeding regime, while 453 genes involved in axon guidance, notch signalling and collagen formation were differentially methylated between cows that received rumen protected methionine and control diet irrespective of their fertility status. Overall, this study indicated that postpartum cows that could potentially become pregnant could be distinguishable based on their endometrial DNA methylation patterns at the time of breeding.

## 1 Introduction

During the early lactation phases of high yielding dairy cows, a higher proportion of nutrition is partitioned to milk production. During this time, the amount of energy spent on milk production and maintenance may not be compensated by feed intake and thus postpartum cows mobilize their body reserves and subsequently enter a state of negative energy balance (NEB). Subsequently, cows exhibit elevated concentrations of ketone bodies, namely beta hydroxybutyrate (ßHB) and non-esterified fatty acids (NEFA) (Butler and Smith, 1989). In turn, the metabolic stress induced during the early lactation period negatively affects the cow’s fertility by altering the transcriptome profile of the maternal environment including endometrium (Wathes et al., 2007; Fenwick et al., 2008; Wathes et al., 2009; Guadagnin et al., 2021) and follicular microenvironment (Llewellyn et al., 2007; Golini et al., 2014; Warma et al., 2020) and miRNAs in follicular fluid (Hailay et al., 2019). Likewise, early lactation-induced metabolic stress reduces fertility by altering the concentration of various reproductive hormones that are controlled by the hypothalamus-pituitary-ovary axis (Beam and Butler, 1999). These may suggest that NEB affects cows’ fertility by altering the transcriptome and/or hormonal profiles that are critical for fertility namely, follicular development, fertilization, and embryonic development. Therefore, identifying maternal molecular signatures associated with the physiological functioning of the reproductive axis might enlighten our understanding of how fertility is regulated by molecular pathways.

Supplementation of dairy cows with rumen-protected essential amino acids such as methionine during the postpartum period has been reported to be an effective strategy to reduce body reserve amino acid mobilization. In this respect, various studies demonstrated positive effects of rumen-protected methionine (RPM) supplementation for improving feed intake (Papas et al., 1984), lactation performance (Overton et al., 1996; Chilliard and Doreau, 1997; Toledo et al., 2017; Wu et al., 2021), fertility (Toledo et al., 2017; Wu et al., 2021) as well as immune responses (Zhou et al., 2016). These beneficial effects are also suggested to be due to increased neutrophil infiltration in the uterus (Stella et al., 2018).

Although it is well known that methionine is involved in the generation of S-adenosylmethionine, a key cofactor in methylation reactions, the molecular mechanism of how RPM supplementation influences the maternal environment is still unclear. Understanding the DNA methylation patterns established in the endometrium during periods of high energy demand with respect to RPM supplementation helps to identify endometrial DNA methylation marks which are directly or directly associated with postpartum dairy cattle fertility. Evidence of altered DNA methylation levels in the decidua of women affected with recurrent pregnancy loss (Yu et al., 2018) and the presence of several differentially methylated genomic regions between proliferative, early and mid-secretory phases in human endometrial cells (Houshdaran et al., 2014) may thereby indicate the feasibility of using DNA methylation marks as reflectors for fertility. In line with this notion, we hypothesize that postpartum dairy cows that are ready for conception and sustain the upcoming pregnancy might be epigenetically distinguishable from those which could fail to conceive and sustain the upcoming pregnancy. In addition, we speculate that supplementation of RPM to the ration of dairy cows during the period of high energy demand might increase the epigenetic divergence between the fertile and non fertile postpartum cows during the time of breeding by modulating the epigenetic profiles of the maternal environment. Therefore, the main objective of the present study was focused to unravel the endometrial epigenetic landscape of postpartum cows during the time of breeding in relation to pregnancy outcomes in the absence or presence of rumen-protected methionine (RPM) supplementation.

## 2 Materials and methods

### 2.1 Animal management and feeding

Detailed information about the experimental animal handling, housing and feeding as well as ethical approval of the experiment was described in a previous publication (Süss et al., 2019). Briefly, the experimental setup and endometrial cytobrush sample collection were conducted by the University of Veterinary Medicine Vienna, Austria. The experiment was conducted on multiparous Holstein-Friesian cows with an average annual energy-corrected milk yield of 9,260 kg. Since the study was conducted on a commercial farm, the number of cows was determined by the total number of multiparous cows calving within 1 year. Furthermore, this study was part of the feeding trail experiment (Süss et al., 2019), the number of postpartum cows used for this part of the study was determined by considering feasibil sample sizes that is appropratie for molecular genetic analysis.

Twelve days post-calving, cows were assigned randomly either to the control (CON) or rumen protected methionine (MET) group. Randomization was performed by building matching pairs based on the expected calving date, parity and previous lactation milk yield. Afterwards, cows of the matched pairs were assigned to CON or MET randomly using the “rand function” of Excel Version 14. Thus, on the farm, cows were assigned to CON or MET groups in an alternative fashion. Since the exact calving might vary in cows within a time window of 2 weeks, allocation to one of these two groups was decided when cows gave birth to a calf. This methodology was chosen to ensure that the same number of cows are within the two groups at any given time and season.

Cows in the CON group received no rumen-protected methionine, whereas cows in the MET groups received daily rumen-protected methionine (RPM) (Mepron^®^, 85% DL-Methionine, Evonik Operations GmbH). For this, at the beginning of the study, a Lys to Met ratio of 3.4:1 was determined in the basal diet using the AminoCowDairy Ration Evaluater, version 3.5.2 (Evonik Industries) and the target was to ensure a Lys to Met ratio to be close to 2.8:1 (Batistel et al., 2017). The calculated amount of RPM per cow ranged between 25.0 and 27.2 g. When considering the mineral and vitamin premix, a concentration of 22.0% ± 3.7% RPM per kg premix was determined. Therefore, when considering the entire study period, the supplemented quantity of RPM was 26.8 ± 4.3 g per cow per day depending on the quantities of the mineral and vitamin premix used.

### 2.2 Endometrial sample collection and classification

**FIGURE 1:**
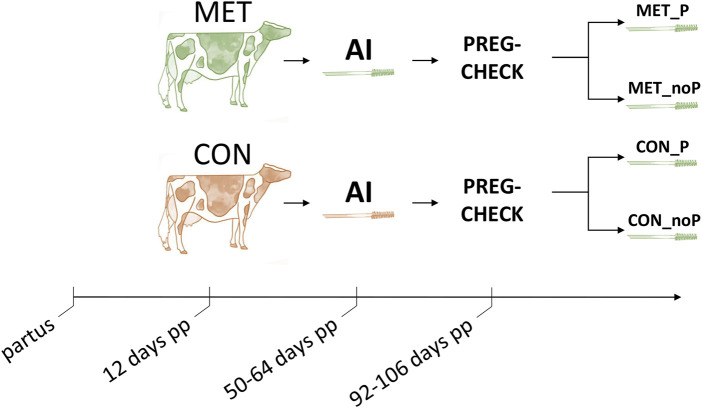
Overall experimental timeline. Multiparous Holstein dairy cows received ether rumen protected methionine (MET, green colour) or a control diet (CON, red colour) starting at day 12 postpartum (pp) until day 100. Cows showing estrus between days 50–64 were artificially inseminated (AI) and an endometrial cytobrush sample was collected 4–8 h later. After pregnancy check, 42 days post breeding, endometrial samples were retrospectively allocated depending on whether they resulted in a pregnancy (MET_P and CON_P) or resulted in no pregnancy (MET_noP and CON_noP). PREG-CHECK; pregnancy check.

Cows showing heat 50–64 days postpartum were subjected to artificial insemination. Endometrial cytobrush samples were collected 4–8 h after insemination. This schedule for sample collection was applied due to the conditions on the farm. The insemination schedule was dependent on whether the cow came to heat and this was monitored by the oestrus detection system. Therefore, endometrial samples were taken shortly after insemination at a defined time (4–8 h after insemination) during estrus time. Once the pregnancy status was determined by ultrasonography 42 days after insemination, the MET and CON groups were classified based on the pregnancy outcome as those that resulted in pregnancy (P) and those that resulted in no pregnancy (noP). Accordingly, endometrial samples collected from cows fed with a control diet that resulted in pregnancy were categorized as control pregnant (CON_P) and those fed a control diet that resulted in no pregnancy were grouped as control non pregnant (CON_noP). Similarly, samples collected from cows supplemented with RPM and resulted in pregnancy were categorized as methionine pregnant (MET_P) and those supplemented with RPM and resulted in no pregnancy were grouped as methionine non pregnant (MET_noP) (Figure 1).

### 2.3 DNA isolation, library preparation and sequencing

Genomic DNA was isolated from 40 endometrial cytobrush samples (CON_P = 8, CON_noP = 12, MET_ P = 8, MET_noP = 12 samples) using AllPrep DNA/RNA/Protein (Qiagen). The concentration and quality of the DNA samples were evaluated using a Nanodrop 8,000 Spectrophotometer (Thermo Fisher Scientific). DNA samples were then subjected to library preparation for reduced representation bisulfite sequencing (RRBS) following a similar method described previously (Zeng et al., 2019). Briefly, 2 µg genomic DNA was digested with MspI enzyme, end-repaired, A-tailed and ligated with methylated adaptor sequences using TruSeq Nano DNA Sample Preparation kit (Illumina, San Diego, CA). Following this, 40–200 bp-sized DNA fragments were extracted using the Zyomclean™ Gel DNA Recovery Kit (Zymo Research). The samples were then treated with bisulfite using the EZ DNA Methylation-Gold Kit™ (Zymo Research). Afterwards, PCR amplification was performed and purified. RRBS libraries were evaluated using the Agilent DNA 1000 kit (Agilent Technologies) and sequenced using Illumina HiSeq2500 at the Research Institute for Farm Animal Biology, Dummerstorf, Germany. The base call output files from sequencing runs were converted into fastq files using the bcl2fastq2 conversion software v2.19. The raw fastq data of 48 samples and the semi processed data are stored in the public repository with GEO accession number GSE248185 and bioproject accession number PRJNA1034815.

### 2.4 Adapter and low quality sequence removal

Various parameters including per base sequence quality, per sequence quality scores, per base sequence content, sequence length distribution, and overrepresented sequences were evaluated using FastQC (Babraham Bioinformatics, UK). Adapter and low-quality sequences were trimmed using Trim Galore (Babraham Bioinformatics, UK) by specifying the RRBS option and discarding reads with <20 nucleotides.

### 2.5 Sequence alignment and extraction methylation calls

Cleaned RRBS sequences were aligned to the bovine reference genome using Bismark Bisulfite Mapper (Babraham Bioinformatics, UK). For this, the bovine reference genome (Bos_taurus.ARS-UCD1.2) was *in vitro* bisulfite converted (C->T and G->A) and indexed using bowtie2-build. Clean reads were aligned to the bisulfite converted reference genome using bowtie2 and the binary alignment map (BAM) files were generated. The BAM files were sorted and sequences from the same library were merged using the SAMtools command (Danecek et al., 2021). Four different BAM files were generated in each of the four experimental groups and methylation calls were obtained from merged BAM files using the Bismark methylation extractor script integrated with the Bismark Bisulfite Mapper (Babraham Bioinformatics, UK). Bismark coverage files were generated and used for downstream analysis.

### 2.6 Quantification of methylation calls and identification of differentially methylated CpG sites

Quantification of the methylation calls and identification of differentially methylated CpG sites (DMCGs) were done using the SeqMonk v1.48.0 tool (Babraham Bioinformatics, UK). In addition to the built-in bovine annotation genome (ARC-USD1.2 v96 assembly), bovine CpG islands, bovine CpG sites, exonic and intronic regions of genes and repetitive elements were imported into the SeqMonk tool. Probe definition was performed using the read position probe generator considering a minimum of 10 read counts per position and 1 valid position per window. Methylation quantitation in each CpG site was performed using bisulfite methylation over features quantification method considering a minimum of 10 counts per CpG site.

Differentially methylation analysis was done using the logistic regression statistical test for the replicates option of the SeqMonk v1.48.0 tool (Babraham Bioinformatics, UK). The methylation level (in percentage) was compared (4 biological replicates in each group) between treatment groups. Adjusted *p*-value cut-off <0.05 was considered for filtering differentially methylated CpG sites (DMCGs). The DMCGs were then annotated with respect to the CpG islands, gene body regions, repetitive elements [short interspersed nuclear elements (SINE), long interspersed nuclear elements (LINE), long terminal repeat elements (LTR), DNA repeat elements (DNA), simple repeats, low complexity repeats, satellite repeats, RNA repeats (rRNA)] and CpG islands, CpG shore, CpG shelf and Open sea.

### 2.7 Molecular pathway analysis

Molecular pathway enrichment analysis was done using g:profiler tool (Raudvere et al., 2019). For this, genes associated with DMCGs were uploaded to g:profiler and *bos taurus* was selected on the organisms’ dropdown menu. Pathway enrichment analysis was performed by considering all bovine gene lists available in the Ensembl database. Bonferroni *p*-value correction was used to identify significant Reactome and KEGG pathways.

## 3 Results

### 3.1 Raw and trimmed RRBS data

Sixteen reduced representation bisulfite sequencing (RRBS) libraries were prepared from four sample groups (CON_P, CON_noP, MET_P, and MET_noP). Each sample group was represented by four libraries. Each RRBS library was sequenced three times. The average bisulfite conversion in CON_P, CON_noP, and MET_P and MET_noP libraries was 98.5, 98.6, 98.6%, and 98.5%, respectively.

A total of 48 raw RRBS sequence data with 125 bp single-end sequences were generated. On average, 13.6, 17.5, 28.7, and 15.0 million reads were generated in each biological replicate of CON_P, CON_noP, MET_P, and MET_noP samples respectively. In terms of the number of bases (10^9^), 1.7, 2.2, 3.6, and 1.8 bases were generated in CON_P, CON_noP, MET_P, and MET_noP samples, respectively. The average GC content of the raw sequence data was 42.3, 41.4, 41.1, and 42.5% in CON_P, CON_noP, MET_P, and MET_noP samples, respectively. After adapter cleaning, 1.1, 1.4, 1.3, and 1.7 Giga base sequences remained for downstream analysis in each biological sample of CON_P, CON_noP, MET_P, and MET_noP samples, respectively. The average GC content of these sequences was 39.5, 38.8, 36.7, and 39.5 in CON_P, CON_noP, MET_P and MET_noP samples, respectively.

### 3.2 Genome-wide endometrial DNA methylation in postpartum cows that resulted in pregnancy and those ended up in no pregnancy

A 24%–35% (on average, 29%) of adapter-cleaned RRBS sequences were uniquely mapped to the bovine reference genome (Bos_taurus.ARS-UCD1.2). On average, 70160823, 97145565, 151232073, and 75931474 of the CpGs sites of the CON_P, CON_noP, MET_P, and MET_noP sample groups, respectively were mapped to 5.9, 6.8, 8.2, and 6.0 million cytosine positions (CpGs) of the bovine reference genome. The global methylation status of each group was evaluated by generating probes using the read position probe generator option of the SeqMonk tool. By considering 10 minimum read counts per position and 1 valid position per window, a total of 5,428,777 CpG sites were identified for downstream analysis. The average global methylation levels were 28.1 ± 6.8, 29.5 ± 6.6, 37.4% ± 2.1%, and 30.8% ± 4.0% in CON_P, CON_noP, MET_P, and MET_noP, respectively. However, after the CpG sites with no valid and zero values were discarded, the number of valid CpG sites was reduced to 199,395. The average methylation levels in these valid CpG sites were 63.5 ± 1.5, 62.5 ± 1.0, 63.9 ± 0.5, and 63.8 ± 1.0% in CON_P, CON_noP, MET_P, and MET_noP, respectively. Although the global average DNA methylation seems to be similar between groups (Supplementary Figure S1), the number of CpG sites with a methylation level >20% was relatively higher in cows that resulted in pregnancy, while the CpG sites with <20% methylation levels tended to be higher in both cows received either RPM supplementation or control diet become no pregnant (Supplementary Figure S1). This in turn suggests the presence of site-specific DNA methylation differences between the cow groups. Since the average global endometrial methylation pattern does not indicate real site-specific methylation differences between groups, we performed differential methylation analysis to identify hypermethylated and hypomethylated DNA methylation marks that could potentially be associated with the fertility of postpartum cows in the presence or absence of RPM supplementation. In the following subsequent sections, hypermethylation and hypomethylation refer to the relative increase and decrease of DNA methylation levels, respectively, in one experimental group compared to the other one.

### 3.3 Endometrial DNA methylation patterns associated with pregnancy establishment in postpartum dairy cows without RPM supplementation

**FIGURE 2:**
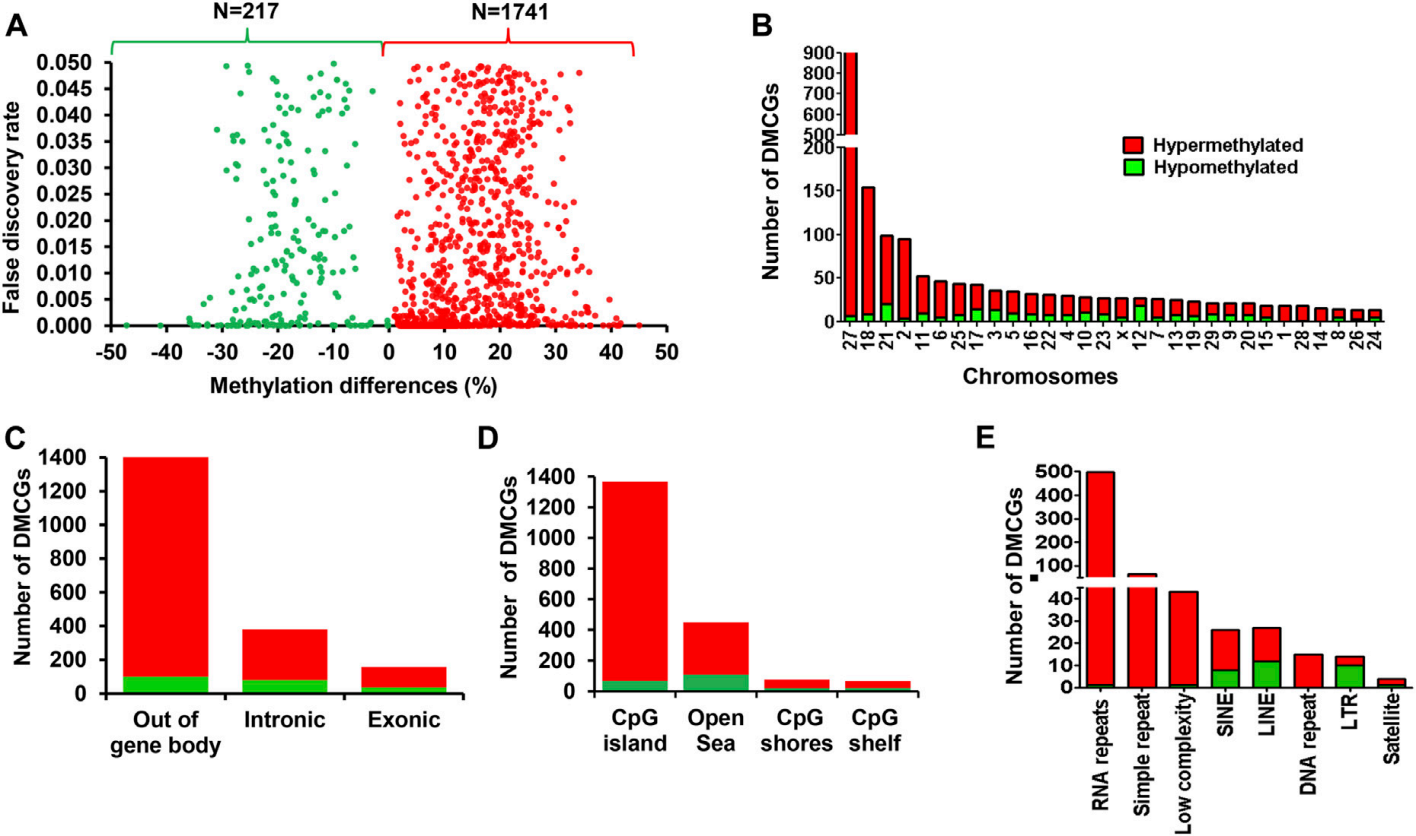
Differentially methylated CpG sites between the CON_P and CON_noP groups. **(A)** Volcano plot showing the methylation levels of hypermethylated CpGs (red dots) and hypomethylated CpGs (green dots) in CON_P compared to the CON_noP group. **(B)** Chromosomal distribution of differentially methylated CpG sites. The number and methylation patterns of differentially methylated CpG sites located on the gene body and out of the gene body regions **(C)**, CpG islands, CpG shores, CpG shelf and Open Sea **(D)** and different types of repetitive elements **(E)**. Red and green dots or bars represent hypermethylated and hypomethylated CpG sites, respectively.

Here we outlined the association between the endometrial DNA methylation landscape and the pregnancy outcomes using endometrial samples that were collected at the time of breeding in cows that resulted in pregnancy (CON_P) or resulted in no pregnancy (CON_noP). We identified a total of 1,958 differentially methylated CpG sites (DMCGs) between these two cow groups. Among these, 88.9% of these CpG sites were found to be hypermethylated and 11.1% were hypomethylated in the CON_P compared to CON_noP cows (Figure 2A). Furthermore, 46.4% (*n* = 908 DMCGs) were located on chromosome 27 (Figure 2B) of which 890 DMCGs were located between 6217254 and 6225600 bp (Supplementary Figure S2).

To gain a deeper insight into the methylation patterns of various genomic features that could potentially be associated with pregnancy outcome, we analysed the DMCGs located on gene body regions, CpG islands and repetitive elements. The results showed that while 27% of these DMCGs were located within gene body regions (exonic and intronic), 73% of the DMCGs were located outside of gene body regions (Figure 2C). With respect to the CpG islands (CGI), 69.8% of DMCGs were located on CGI and 22.9, 3.8%, and 3.4% of DMCGs were located on Open Sea, CpG shore and CpG shelf, respectively (Figure 2D). Among those located on CGI, a total of 892, 108, 79, and 58 DMCGs overlapped with four CGIs, namely oe = 1.12 (chr27:6217254–6225600 bp), oe = 1.08 (chr18:59225247–59226010 bp), oe = 1.13 (chr2:121571599–121575924 bp) and oe = 0.78 (chr21:33002605–3302896 bp), respectively (Supplementary Table S1). In addition, 675 DMCGs were overlapped with different classes of repetitive elements (RE), and 98%–100% of DMCGs overlapped with rRNA repetitive elements, simple repeats, low complexity and DNA repeats and 56%–75% of the DMCGs overlapped with LINE, SINE and satellite were hypermethylated in CON_P compared to CON_noP cows. In contrast, the proportion of hypomethylated DMCGs overlapped on the LTR outpaced the hypermethylated ones (Figure 2E).

### 3.4 Genes associated with differentially methylated CpG sites between cows that resulted in pregnancy and no pregnancy without RPM supplementation

**FIGURE 3:**
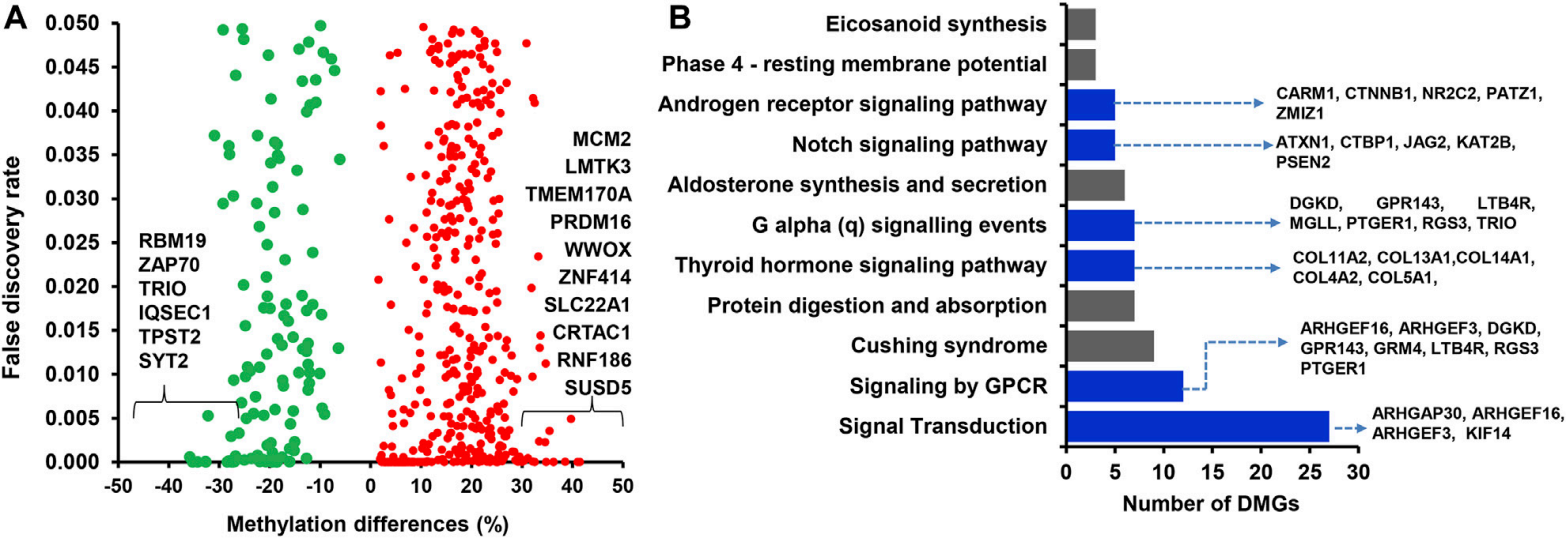
**(A)** Volcano plot displaying the methylation levels of hypermethylated (red dot) and hypomethylated (green dots) CpG sites associated with annotated genes in CON_P compared to the CON_noP group. Red and green dots represent hypermethylated and hypomethylated CpG sites, respectively. **(B)** Functional annotation of genes associated with differentially methylated CpG sites between CON_P and CON_noP. Blue bars indicate the signalling pathways. DMGs; Differentially methylated genes.

Among the 1,958 DMCGs identified in CON_P vs. CON_noP groups, 537 DMCGs were overlapped with 313 annotated genes (Figure 3A; Supplementary Table S2). Pathway analysis indicted those genes associated with either hypermethylated or hypomethylated CpG sites was involved in various signaling pathways including signal transduction, signalling by GPCR, G alpha (q) signalling events and other pathways including Cushing syndrome, alderstonen synthesis and secretion and eicosanoid pathways (Figure 3B; Supplementary Table S3). Moreover, among differentially methylated genes, methylation level of *LMTK3, TMEM170A, PRDM16, ZNF414, SLC22A1, CRTAC1, RNF186, SUSD5,* and *ANKRD53* was significantly increased (33%–41%), while the methylation level of *RBM19, TPST2,* and *SYT2* genes was significantly decreased (32.2%–35.8%) in the CON_P compared to CON_noP groups (Table 1; Supplementary Table S2). In addition, 76 of the 305 differentially methylated genes were differentially methylated in ≥2 CpG sites. For instance, the gene that is associated with a novel transcript (*ENSBTAT00000081135.1*) was Differntially methylated in 62 CpG sites. Similarly, *RF00002 (*5.8S ribosomal RNA), *ADAP1, ZNF75D, RSPH6A, KLHL35, ENTPD8, DPP6,* and *ABLIM2* were differentially methylted in 5 to 13 CpG sites (Supplementary Table S2).

**TABLE 1:** Differentially methylated genes with ≥30% difference between CON_P and CON_noP.

Symbol	Description	Differences (%)	FDR
*LMTK3*	Lemur tyrosine kinase 3	41.0	0.000
*TMEM170A*	Transmembrane protein 170A	39.7	0.005
*PRDM16*	PR/SET domain 16	38.6	0.000
*ZNF414*	Zinc finger protein 414	35.5	0.004
*SLC22A1*	Solute carrier family 22 member 1	34.7	0.011
*CRTAC1*	Cartilage acidic protein 1	34.6	0.002
*RNF186*	Ring Finger Protein 186	33.7	0.014
*SUSD5*	Sushi domain containing 5	33.4	0.002
*ANKRD53*	Ankyrin repeat domain 53	33.3	0.023
*ARHGEF3*	Rho guanine nucleotide exchange factor 3	32.6	0.000
*CASZ1*	Castor zinc finger 1	32.5	0.041
*UROS*	Uroporphyrinogen III synthase	32.2	0.000
*ZNF775*	Zinc finger protein 775	32.2	0.041
*CARM1*	Coactivator associated arginine methyltransferase 1	32.1	0.010
*ANKRD53*	Ankyrin repeat domain 53	30.9	0.048
*DUSP18*	Dual specificity phosphatase 18	30.5	0.000
*AFF3*	Af4/fmr2 family member 3	30.3	0.000
*SYT2*	Synaptotagmin 2	−32.2	0.005
*TPST2*	Tyrosylprotein sulfotransferase 2	−32.6	0.000
*RBM19*	RNA binding motif protein 19	−35.8	0.001

### 3.5 Endometrial DNA methylation patterns in postpartum cows that resulted in pregnancy and no pregnancy after RPM supplementation

**FIGURE 4:**
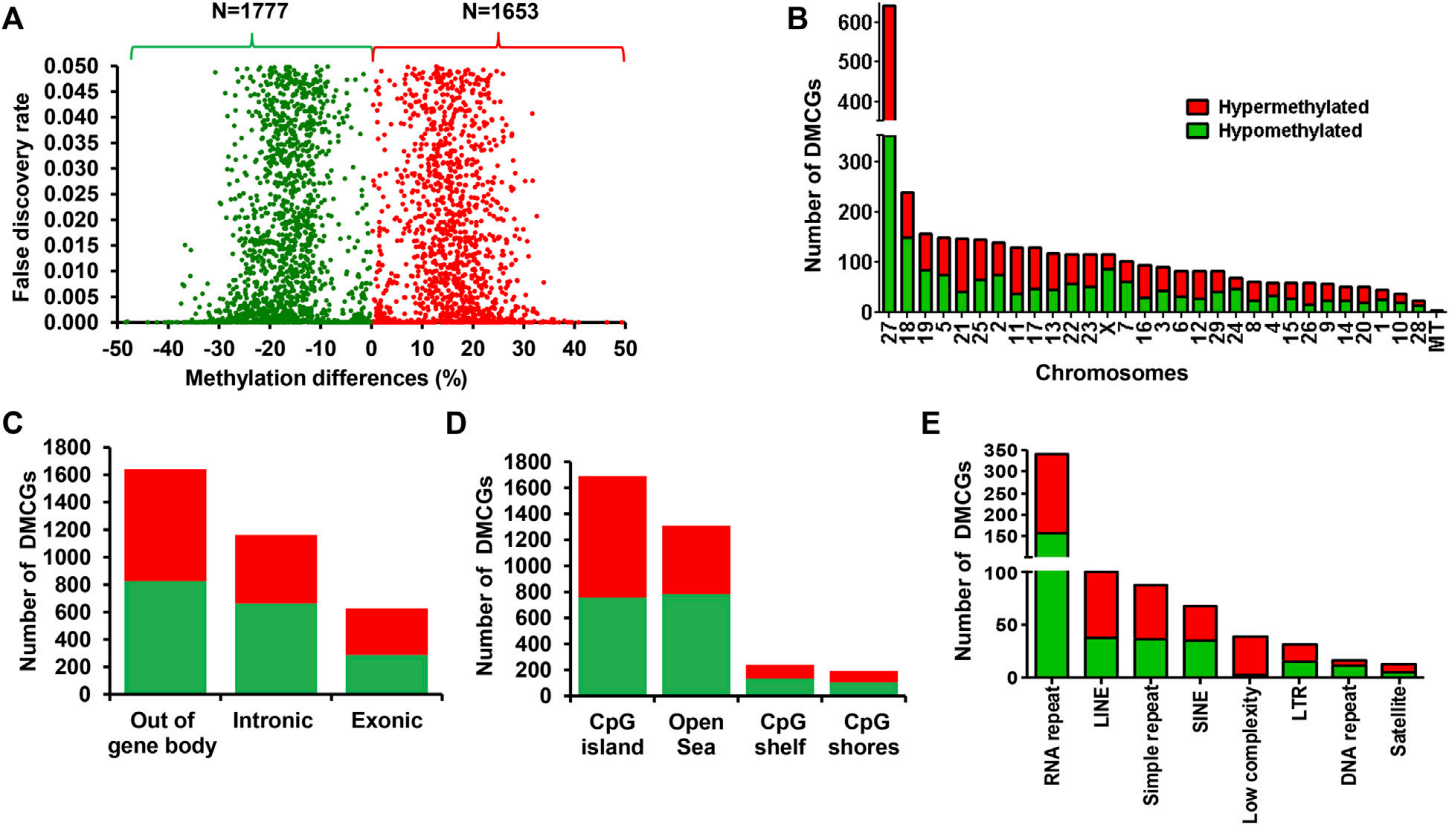
Differentially methylated CpG sites between the MET_P and MET_noP groups. **(A)** Volcano plot displaying the hypermethylated (red dots) and hypomethylated CpG sites (green dots) in MET_P compared to the MET_noP group. **(B)** Chromosomal distribution of differentially methylated CpG sites. The number and methylation patterns of differentially methylated CpG sites located on the gene body and out of the gene body regions **(C)**, CpG islands, CpG shores, CpG shelf and Open Sea **(D)** and different types of repetitive elements **(E)**. Red and green dots or bars represent hypermethylated and hypomethylated CpG sites, respectively.

To clarify the effect of methionine supplementation on the endometrial epigenome profile in relation to pregnancy outcome, we investigated the endometrial DNA methylome profile of postpartum dairy cows supplemented with RPM that subsequently resulted in pregnancy (MET_P) or resulted in no pregnancy (MET_noP). In this regard, 3,430 DMCGs were identified, of which 48.2% (*n* = 1,653) and 51.8% (*n* = 1,777) were hypermethylated and hypomethylated, respectively in MET_P compared to MET_noP cows (Figure 4A). These DMCGs were distributed over all chromosomes (Figure 4B), but relatively a higher proportion (*n* = 592, 18.7%) of DMCGs were located on chr27:6217254–6225600 bp. The proportion of hypermethylated DMCGs outpaced the proportion of hypomethylated ones by more than two folds on chromosomes, 26, 21, 16, 12, and 11. Conversely, on chromosomes X, 18, 24, and 28, the hypomethylated DMCGs outpaced the hypermethylated ones by more than two folds (Figure 4B).

Further analysis of the DMCGs with respect to overlapping genes indicated that 52% of these DMCGs were located in gene body regions, while 48% were located out of gene body regions (Figure 4C). A closer inspection into these gene body methylation patterns revealed relatively higher numbers of hypomethylated CpG sites within intronic regions, whereas the proportion of hypermethylated and hypomethylated CpG sites was nearly equal for DMCGs located within exonic regions or off the gene body regions. Similarly, analysis with the context of CpG islands (CGI), CpG shore, CpG shelf and open sea regions (Figure 4D) indicated that 49.2% (*n* = 1,690) of DMCGs were overlapped with CGI. Of these 594, 85 and 74 DMCGs were overlapped with oe = 1.12 (chr27: 6217254–6225600), oe = 1.08 (ch18: 59225247–59226010) and oe = 1.13 (ch2: 121571592–121575924) CpG islands, respectively (Supplementary Table S4). In addition to CGI, 698 DMCGs were found to be mapped to different classes of RE (Figure 4E).

### 3.6 Differentially methylated genes between postpartum cows that resulted in pregnancy and no pregnancy after RPM supplementation

**FIGURE 5:**
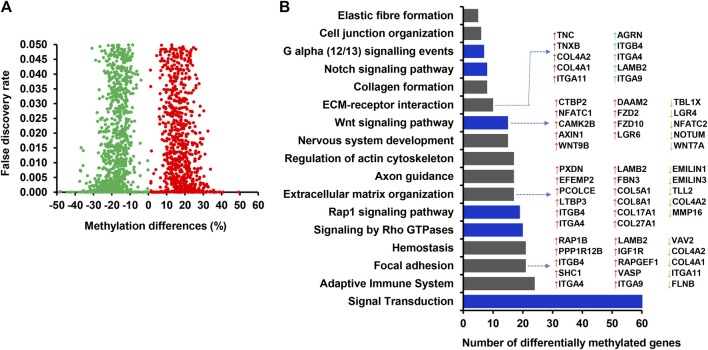
**(A)** Volcano plot showing the number of hypermethylated (red dot) and hypomethylated (green dots) CpG sites associated with annotated genes in MET_P compared to MET_noP. **(B)** Functional annotation of differentially methylated genes identified in MET_P vs. MET_noP. Blue bars indicated signalling pathways. Red and green dots represent hypermethylated and hypomethylated CpG sites, respectively. Arrows: ↑ and ↓ on the left side of the gene symbols indicate hypermethylation and hypomethylation, respectively.

Among 3,430 differentially methylated CpG sites between the MET_P and MET_noP cows, 1,781 DMCGs were overlapped with 890 genes (Figure 5A; Supplementary Table S5). Of these, the methylation level of *AFF3, VOPP1, ERG, SLC2A11, PXDN, DTX4,* and *BMP2K* genes was increased by 35%–49% while the methylation level of genes including C*CDC151, CDC42BPA, FLNB, SLC4A3, TPO,* and *TRAF3IP1* was decreased by 35%–48% in MET_P compared to the MET_noP cows (Table 2). Considering the frequency of DMCGs, 325 genes exhibited differential methylation in more than 2 CpG sites (Supplementary Table S5). For instance, the methylation pattern of *SPRED3, RAP1B, BCR, SBK1* and *IFFO1* was increased in 10–27 CpG sites. Vice versa, the methylation level of genes including, *RF00001, UBE2E20, PRAME, FBXO41,* and *TSHZ2* was decreased in 8–10 CpG sites (Supplementary Table S5). Interestingly, genes associated with either hypermethylated or hypomethylated DMCGs were subjected to pathway analysis. These genes are involved in various key developmental and signal pathways including signal transduction, Rap1 signalling, WNT-signalling, focal adhesion, ECM receptor interaction, Hemostasis and collagen formation (Figure 5B; Supplementary Table S6).

**TABLE 2:** Differentially methylated genes with ≥30% increase or decrease in MET_P compared to MET_noP.

Gene symbol	Description	Methylation differences (%)	FDR
*AFF3*	AF4/FMR2 family member 3	49.3	0.000
*SLC2A11*	Solute carrier family 2 member 11	40.8	0.000
*DTX4*	Deltex e3 ubiquitin ligase 4	39.2	0.000
*BMP2K*	BMP2 inducible kinase	38.7	0.000
*VOPP1*	VOPP1, WBP1/VOPP1 family member	37.0	0.000
*PXDN*	Peroxidasin	35.7	0.000
*ERG*	ETS transcription factor ERG	35.1	0.000
*EGR3*	Early growth response 3	33.9	0.000
*NUBP2*	Nucleotide binding protein 2	33.8	0.000
*APLN*	apelin	32.7	0.002
*EFEMP2*	EGF containing fibulin extracellular matrix protein 2	32.6	0.021
*DST*	Dystonin	32.1	0.000
*ASB1*	Ankyrin repeat and SOCS box containing 1	−30.1	0.000
*CNIH3*	Cornichon family AMPA receptor auxiliary protein 3	−30.7	0.049
*PRG3*	Proteoglycan 3	−30.8	0.000
*MXRA5*	Matrix remodeling associated 5	−30.8	0.000
*PASK*	PAS domain containing serine/threonine kinase	−30.9	0.000
*EEPD1*	Endonuclease/exonuclease/phosphatase family domain containing 1	−31.2	0.001
*RTL6*	Retrotransposon gag like 6	−31.3	0.000
*TPCN1*	Two pore segment channel 1	−31.6	0.000
*ITGA11*	Integrin subunit alpha 11	−31.8	0.003
*RNF39*	Ring finger protein 39	−31.8	0.001
*PACS2*	Phosphofurin acidic cluster sorting protein 2	−32	0.002
*SMTN*	Smoothelin	−32.9	0.000
*THAP4*	THAP domain containing 4	−33.8	0.000
*OSBPL10*	Oxysterol binding protein like 10	−33.9	0.000
*MMP16*	Matrix metallopeptidase 16	−34.2	0.000
*TEAD4*	TEA domain transcription factor 4	−34.6	0.000
*SLC4A3*	Solute carrier family 4 member 3	−35.3	0.001
*TRAF3IP1*	TRAF3 interacting protein 1	−35.5	0.014
*FAM20C*	FAM20C, golgi associated secretory pathway kinase	−36.6	0.000
*FLNB*	Filamin B	−36.7	0.000
*CCDC151*	Coiled-coil domain containing 151	−37.1	0.007
*ENO1*	Enolase 1	−40.6	0.000
*TPO*	Thyroid peroxidase	−42.2	0.000
*CDC42BPA*	CDC42 binding protein kinase alpha	−48.4	0.000

### 3.7 Endometrial DNA methylation patterns associated with pregnancy establishment in postpartum cows fed with a control diet and supplemented with RPM

**FIGURE 6:**
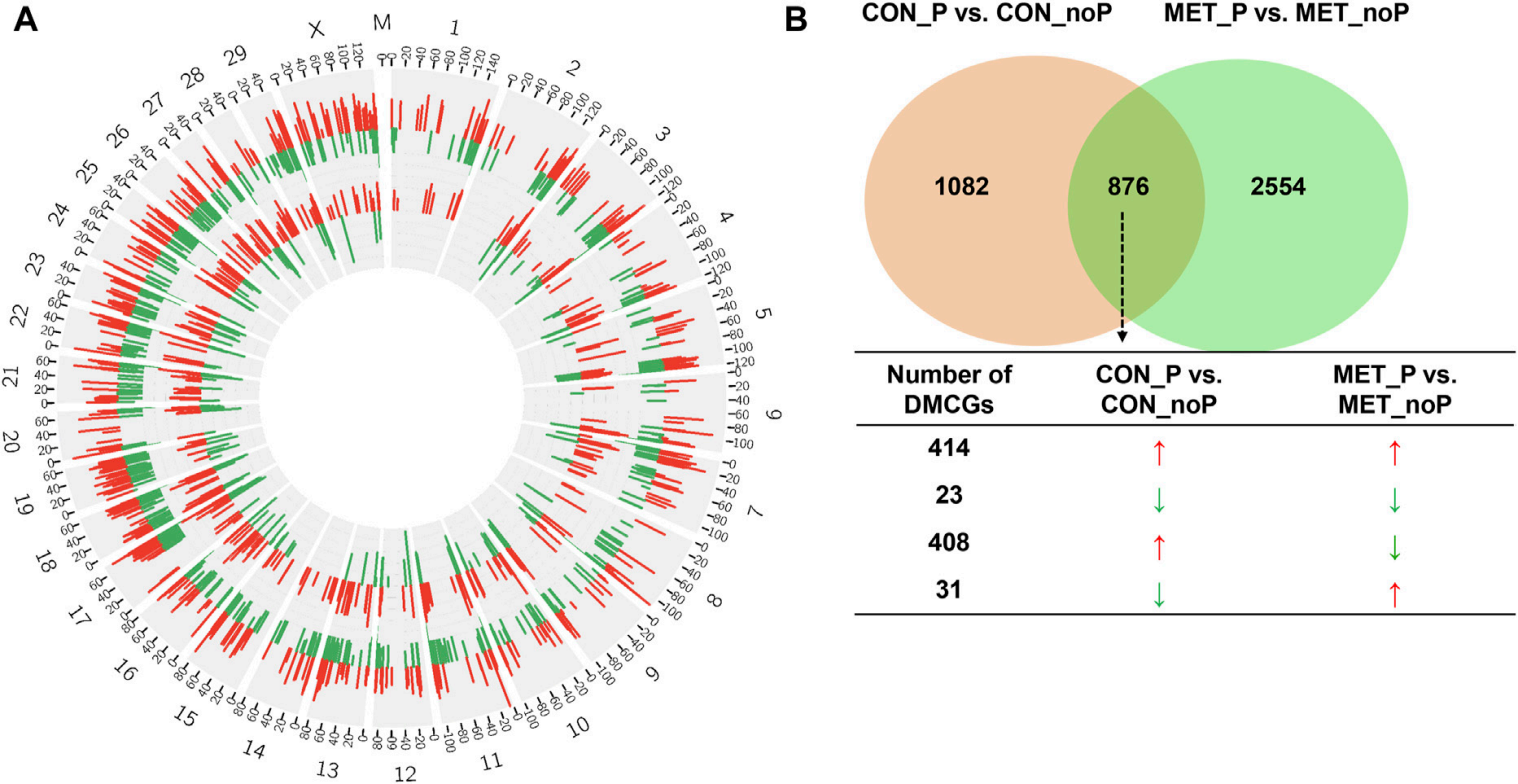
**(A)** Circos plot displaying the chromosomal distribution of differentially methylated CpG sites between MET_P vs. MET_noP (outer circos) and CON_P vs. CON_noP (inner circos). Numbers 1–29, X and M indicate autosomal, X and mitochondrial chromosomes, respectively. Red and green bars represent hypermethylated and hypomethylated CpG sites, respectively in MET_P compared to MET_noP as well as in CON_P compared to CON_noP. **(B)** Exclusively and commonly differentially methylated CpG sites in CON_P vs. CON_noP and MET_P vs. MET_noP. Arrows: ↑ and ↓ on indicate the hypermethylation and hypomethylation, respectively. Circos plots were generated using the circos software (http://circos.ca/software/download/circos/), licensed under GNU General Public License (GPL) v3 (Krzywinski, M. et al. 2009).

**FIGURE 7:**
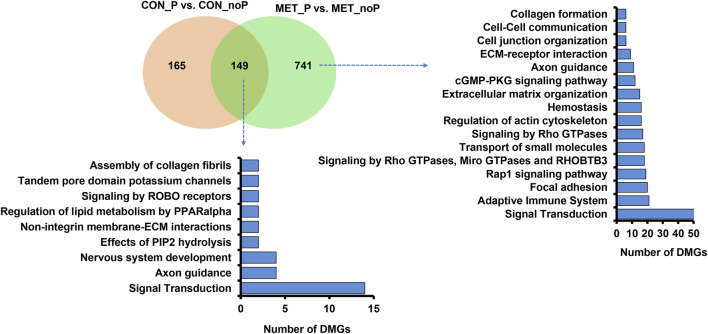
In common and exclusively differentially methylated genes in CON_P vs. CON_noP and MET_P vs. MET_noP groups (left) and their functional annotations (right).

Once we investigated the endometrial DNA methylation landscape in relation to subsequent pregnancy outcomes in cows fed a control diet (CON_P vs. CON_noP) or received RPM twice daily (MET_P vs. MET_noP), we analysed the common DMCGs in both comparisons. With this respect, the methylation profiles plotted in 20 Mbp windows showed the presence of common DMCGs in cows that resulted in pregnancy compared to those that resulted in no pregnancy in both feeding groups (Figure 6A). Interestingly, 44.7% of DMCGs identified in CON_P vs. CON_noP were also differentially methylated between the MET_P and MET_noP cows. Likewise, 25.6% of DMCGs identified in MET_P vs. MET_noP cows were also differentially methylated between CON_P and CON_noP cows (Figure 6B). Of commonly detected DMCGS, 414 and 23 were hypermethylated and hypomethylated in both CON_P and MET_P cows, respectively compared to their counterparts. Interestingly, 289 DMGCs were localized only on chromosome 27 (Supplementary Figure S3) between 6,219.5 and 6,225.2 kb (Supplementary Figure S4). With respect to genes, we identified 149 commonly differentially methylated genes between the CON_P and CON_noP cows as well as between the MET_P and MET_noP cows (Figure 7; Supplementary Table S7). Of these, 45 genes including *ABLIM2, AFF3, AGO2, BMP6, CDC88C,* and *CDK5RAP2* were hypermethylated, while 33 genes including *COL4A2, NAXD, INTS1, JAG2,* and *LRRC55* were hypomethylated (Table 3; Supplementary Table S7) in both CON_P and MET_P.

**TABLE 3:** Genes with increased methylation levels in CON_P compared to CON_noP as well as MET_P compared to MET_noP.

	CON_P vs. CON_noP		MET_P vs. MET_noP	
Gene symbol	Differences (%)	FDR	Differences (%)	FDR
*ABLIM2*	14.6	0.037	21.8	0.044
*AFF3*	30.3	0.000	49.3	0.000
*AGO2*	17.4	0.044	28.1	0.000
*BMP6*	16.9	0.001	12.0	0.005
*CACNA1H*	18.8	0.009	14.2	0.039
*CAMK2B*	28.7	0.000	24.2	0.000
*CARM1*	31.9	0.020	25.5	0.011
*CASZ1*	32.5	0.041	15.9	0.024
*CCDC88C*	25.1	0.006	13.7	0.035
*CDK5RAP2*	11.1	0.028	10.7	0.001
*CHCHD6*	19.4	0.038	8.7	0.004
*COL5A1*	22.3	0.045	16.7	0.008
*CROCC*	18.9	0.045	16.5	0.011
*CUX1*	25.4	0.006	8.1	0.024
*DEPDC5*	21.1	0.000	15.0	0.005
*FNDC1*	21.8	0.000	14.4	0.008
*FOXK1*	11.0	0.033	12.8	0.003
*FSCN2*	17.7	0.028	18.0	0.026
*GABRA5*	16.8	0.026	17.6	0.030
*GGT1*	13.9	0.030	15.9	0.027
*GLCCI1*	21.0	0.000	9.4	0.001
*GLG1*	24.3	0.028	12.0	0.008
*ICE1*	16.2	0.049	12.3	0.048
*KAT2B*	23.7	0.000	9.7	0.002
*LY86*	21.1	0.013	17.1	0.002
*MARCH8*	24.0	0.003	9.6	0.028
*MGLL*	21.1	0.008	8.7	0.017
*NFIX*	24.3	0.026	20.5	0.017
*PES1*	19.7	0.021	23.1	0.048
*PPM1H*	19.4	0.032	23.4	0.000
*PRAME*	19.7	0.031	19.7	0.004
*PTPN7*	23.8	0.041	26.7	0.016
*RAI1*	14.7	0.028	15.8	0.039
*RGS3*	19.7	0.017	25.5	0.001
*SEPT9*	16.3	0.027	28.7	0.008
*TEP1*	21.5	0.020	13.8	0.000
*TTC7B*	23.2	0.000	17.7	0.000
*WWOX*	33.5	0.013	24.4	0.000
*COL4A2*	−20.6	0.021	−27.0	0.005
*INTS1*	−13.4	0.029	−9.6	0.019
*JAG2*	−20.5	0.002	−12.4	0.021
*LRRC55*	−16.7	0.018	−13.7	0.042
*NAXD*	−16.0	0.000	−16.6	0.000
*PPIL2*	−14.5	0.033	−18.4	0.033
*PTPRN2*	−18.5	0.036	−13.7	0.006
*RARB*	−28.4	0.000	−21.9	0.016
*STARD10*	−14.2	0.047	−13.0	0.019
*TBL1X*	−25.0	0.000	−1.8	0.000

### 3.8 Exclusively differentially methylated CpG sites in postpartum cows fed with a control diet or supplemented with rumen-protected methionine

Despite the presence of common DMCGs, 55.3% of DMCGs identified in CON_P vs. CON_noP cow groups were not differentially methylated in MET_P vs. MET_noP cows. Likewise, 74.6% of DMCGs identified in MET_P vs. MET_noP cows were not differentially methylated in CON_P vs. CON_noP cows (Figure 6B). When the analysis was narrowed down to the gene level, the methylation patterns of the CpG sites overlapped with 165 genes were significantly altered exclusively in CON_noP compared to CON_P cows but not in the MET_noP compared to the MET_P cows (Figure 7; Supplementary Table S8). Conversely, 741 genes differentially methylated between MET_P and MET_noP cows were not significantly differentially methylated between CON_P and CON_noP cows (Figure 7; Supplementary Table S9). Among these exclusively differentially methylated genes between MET_P and MET_noP cows, the methylation level of several sets of gene families including collagens (*COL17A1, 8A1*), insulin-like growth factors (*IGF1R, 2R*), interleukins (*IL17RB, REL, IL34, IL4I1*), integrins (*ITGA4, A9, B4*, and *BL1*), potassium voltage-gated channels (*KCNB1, H2, J6, K17*, and *Q1*), myosin heavy chains (*MYH10, 11, 14, 18B, 7A*, and *9B*), protein tyrosine phosphatases (*PTP4A3, N21, RC, RE*), solute carrier (*SLC12A5, 29A1, 2A11, 34A3, 41A3, 43A1, 45A4, 4A2, 6A12*, and *7A5*) and zinc finger proteins (*ZNF521, 24, 618, 704*) was increased in MET_p group. On the contrary, the methylation level of distinct gene families including adhesion G protein-coupled receptors *(ADGRA1, B1*, and *G1),* Rho GTPase activating proteins (*ARHGA15, 22*, and *45),* bromodomains *(BRD1, 3),* families with sequence similarity (*FAM107B, 163B, 20C, 222A, 234A, 53B*, and *83E),* forkhead box genes *(FOXK2, N1, N3),* heat shock proteins *(HSPA1A, B7),* protein tyrosine phosphatase receptors *(PTPRF, PTPRS)* solute carrier *(SLC16A5,19A3, 20A2, 39A11, 4A3, 8A2*, and *8A3),* transmembrane proteins *(TMEM184A, 229B, 232, 240*, and *43*), zinc finger proteins *(ZNF423, 516, 771, 787*, and *862)* and prostaglandin I2 receptors *(PTGIR)* was decreased in MET_P compared to MET_noP (Supplementary Table S9). Pathway analysis showed that these differentially methylated genes specific for MET_P vs. MET_noP cows are involved in various pathways including focal adhesion, extracellular matrix organization, collagen formation and cell-cell communication (Figure 7).

### 3.9 Effect of RPM supplementation on the endometrial DNA methylation patterns of postpartum cows that resulted in pregnancy

**FIGURE 8:**
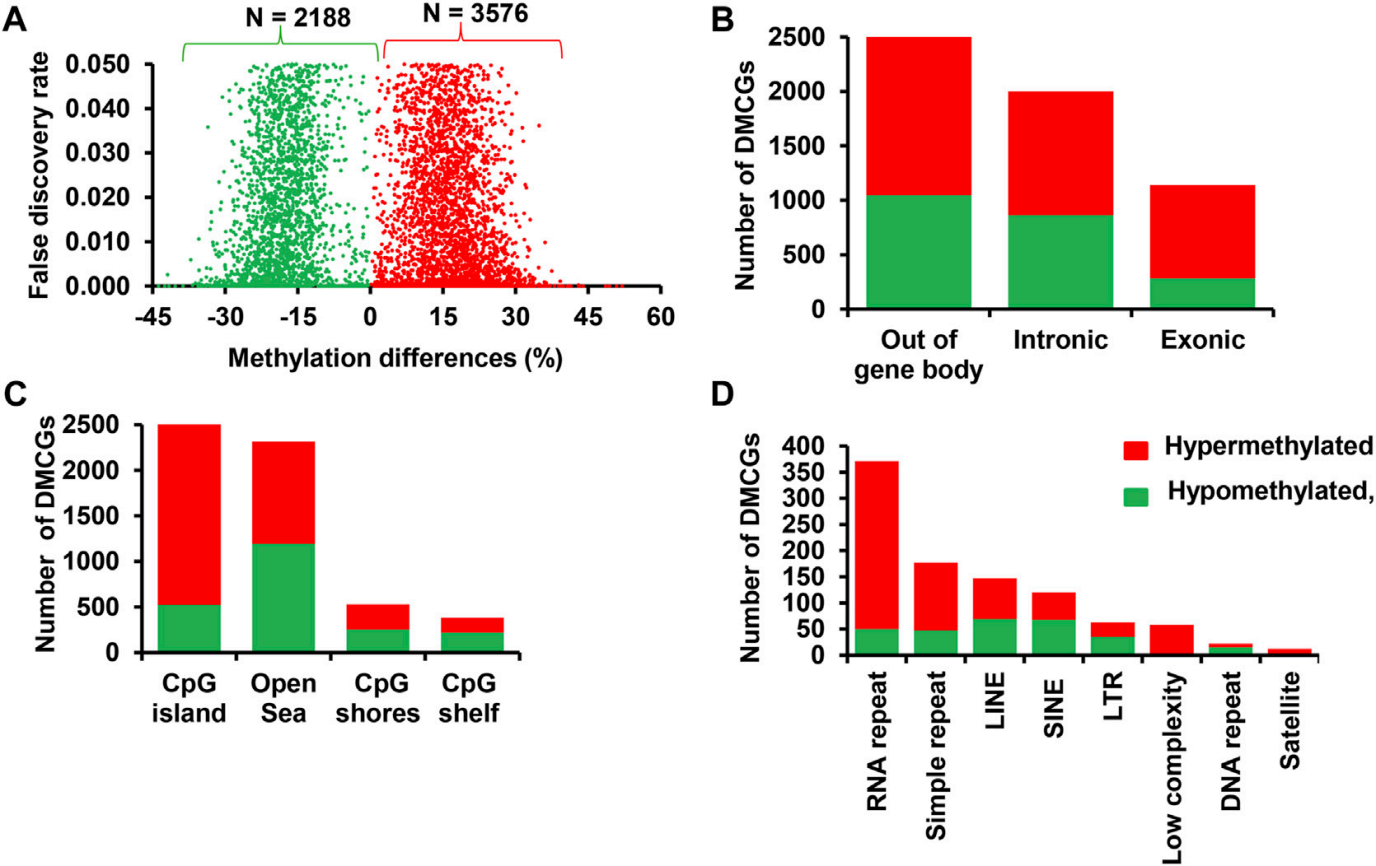
Differentially methylated CpG sites between MET_P and CON_P. **(A)** Volcano plot displaying the methylation level of hypermethylated (red dots) and hypomethylated CpG sites (green dots) in MET_P compared to the CON_P group. Differentially methylated CpG sites located on the gene body and out of the gene body regions **(B)**, CpG islands, CpG shores, CpG shelf and Open Sea **(C)** and different types of repetitive elements **(D)**. Red and green dots or bars represent hypermethylated and hypomethylated CpG sites, respectively.

The number of DMCGs and differentially methylated genes between pregnant cows and those ending up with no pregnancy was relatively higher in those supplemented with RPM compared to the control diet group. Therefore, to test whether supplementation of RPM affects the endometrial epigenome profile of postpartum cows in a fertility-dependent manner during the breeding time, the DNA methylation patterns of cows that received RPM and resulted in pregnancy (MET_P) were compared with cows that resulted in pregnancy but received only the control diet (CON_P). The results revealed that 5,764 CpG sites were differentially methylated between the two groups. Of these, 3,576 and 2,188 DMCGs were hypermethylated and hypomethylated in MET_P compared to CON_P cows, respectively (Figure 8A). Further characterization of these DMCGs according to their genomic structures indicated that 54.5% were localized in gene body regions and 63.6% of the DMCGs located in the gene body regions were hypermethylated in MET_P compared to CON_P cows (Figure 8B).

Concerning the genes, 3,126 DMCGs were overlapped with 1,318 genes including transmembrane proteins, solute carriers, ATPases, calcium voltage-gated channel subunits, myosins, prostaglandins, potassium voltage-gated channels, and G protein-coupled receptors (Table 4; Supplementary Table S10). A total of 539 genes were differentially methylated at ≥ 2 CpG sites (Supplementary Table S11). Among these, *MTCL1, FHDC1, SPRED3, ATP8B, RAP1B, GPR62* and *SOX9* genes were hypermethylated at 16–34 CpG sites, while others including *ADGRB1, UBE2E2* and *TSHZ2* were hypomethylated at 17, 14, and 12 CpG sites, respectively (Supplementary Table S11).

**TABLE 4:** Differentially methylated family of genes between MET_P and CON_P groups.

Family of genes	Symbol	Methylation status	Family of genes	Symbol	Methylation status
Rho GTPase activating proteins	*ARHGAP23*	↑	Solute carrier family	*SLC22A16*	↑
	*ARHGAP27*	↑		*SLC22A31*	↑
	*ARHGEF10L*	↑		*SLC25A5*	↑
	*ARHGEF19*	↑		*SLC29A3*	↑
	*ARHGEF7*	↑		*SLC39A11*	↑
	*ARHGAP25*	↓		*SLC41A3*	↑
	*ARHGAP32*	↓		*SLC43A1*	↑
	*ARHGEF16*	↓		*SLC45A4*	↑
	*ARHGEF2*	↓		*SLC49A3*	↑
	*ARHGEF3*	↓		*SLC13A5*	↓
	*ARHGEF4*	↓		*SLC20A2*	↓
ATPases	*ATP11A*	↑		*SLC25A42*	↓
	*ATP1A3*	↑		*SLC4A3*	↓
	*ATP2B2*	↑	Potasium voltage-gated channel subfamily	*KCNA1*	↓
	*ATP8B1*	↑		*KCNK13*	↓
	*ATP9A*	↑		*KCNK5*	↓
	*ATP13A2*	↓	Regulator of G protein signaling	*RGS3*	↓
	*ATP1B1*	↓		*RGS6*	↓
	*ATP2B4*	↓		*RGS7*	↓
	*ATP6V0D2*	↓	Mitogen-activated protein kinase	*MAP2K6*	↑
Calcium voltage-gated channel subunits	*CACNA1C*	↑		*MAP3K15*	↑
	*CACNA1I*	↑		*MAP7D1*	↑
	*CACNG5*	↑		*MAPK8IP3*	↑
G protein-coupled receptors	*GPR26*	↑	Myosins	*MYH11*	↑
	*GPR37L1*	↑		*MYH14*	↑
	*GPR50*	↑		*MYLK4*	↑
				*MYBPC2*	↑
	*GPR62*	↑	Transmembrane protein	*TMEM132A*	↑
	*GPRC5C*	↑		*TMEM145*	↑
Protein phosphatase	*PPP1R18*	↑		*TMEM169*	↑
	*PPP1R3F*	↑		*TMEM170B*	↑
Prostaglandins	*PTGIR*	↑		*TMEM184A*	↑
	*PTGIS*	↑		*TMEM200C*	↑
	*PTGS1*	↑		*TMEM86B*	↑
				*TMEM88*	↑

Arrows, ↑ and ↓ indicate increased and decreased methylation patterns, respectively in the MET_P cows group compared to CON_P.

Apart from the gene body regions, 79.3% of DMCGs were mapped to CpG islands and 70% were mapped to different classes of repeats with the majority of them found to be hypermethylated in MET_P compared to CON_P cows (Figures 8C, D). Thus, compared to the control diet, RPM supplementation caused increased methylation of exonic, intronic, CpG islands and different classes of repeats in the endometrial cells of cows that resulted in pregnancy following artificial insemination.

### 3.10 Effects of RPM supplementation on the endometrial DNA methylation patterns of postpartum cows that resulted in no pregnancy

**FIGURE 9:**
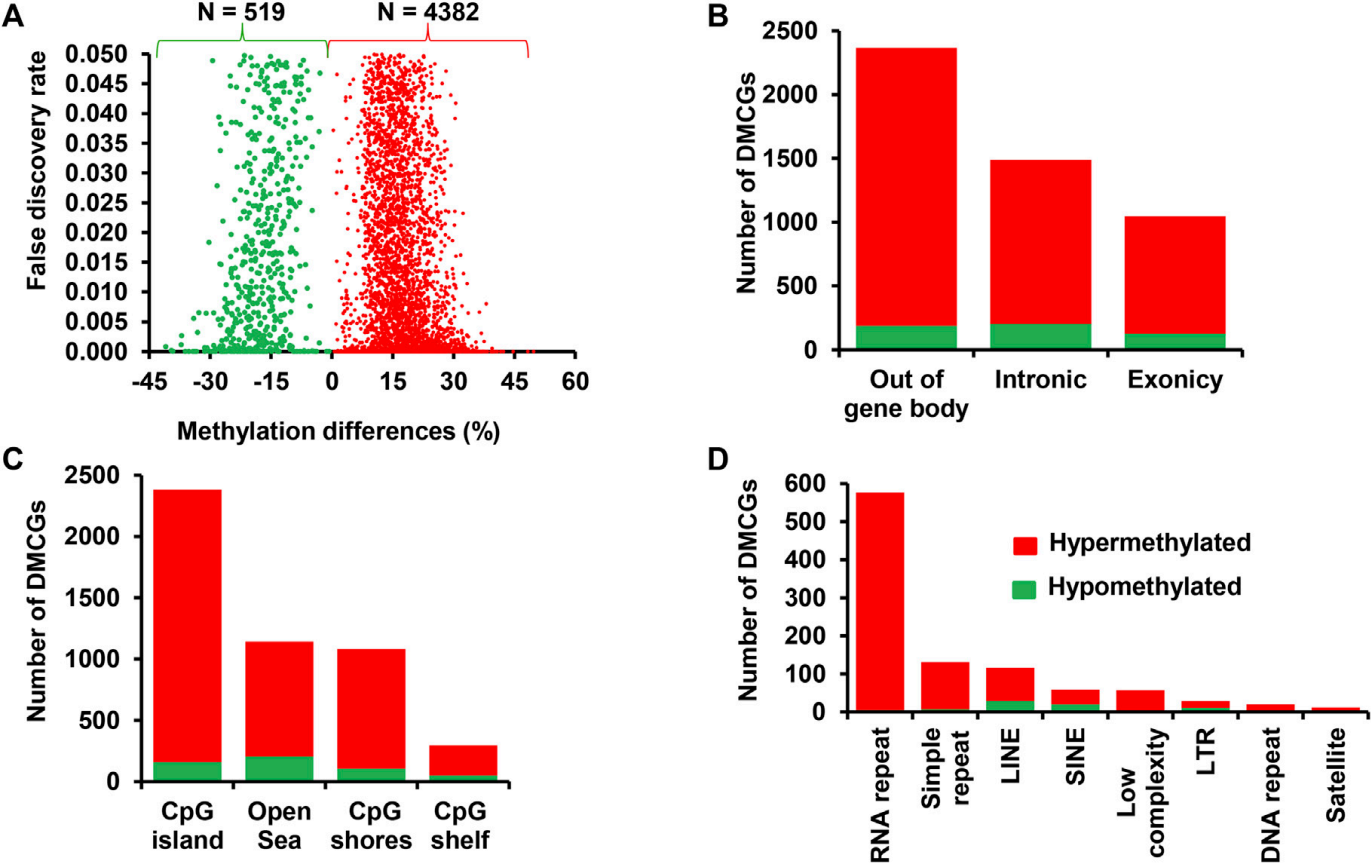
**(A)** Differentially methylated CpG sites between MET_noP and CON_noP: Volcano plot showing the number of hypermethylated (red dots) and hypomethylated CpG sites (green dots) in MET_noP compared to the CON_noP group. Differentially methylated CpG sites located on the gene body and out of the gene body regions **(B)**, CpG islands, CpG shores, CpG shelf and Open Sea **(C)** and different types of repetitive elements **(D)**. Red and green dots or bars represent hypermethylated and hypomethylated CpG sites, respectively.

The effect of supplementation of RPM on the endometrial epigenome profile of postpartum dairy cows that resulted in no pregnancy was analysed by comparing the DNA methylation levels of the MET_noP and CON_noP groups. This analysis revealed 4,901 DMCGs between these groups of which 89.5% showed a higher methylation level in MET_noP cows (Figure 9A). Furthermore, 69.7% of the DMCGs were located within gene body regions (Figure 9B). Of those found in the gene body regions, 2,521 DMCGs were overlapped with 1,062 genes including forkhead boxes*,* transmembrane proteins, solute carriers, ATPases, calcium voltage-gated channel subunits, myosins, prostaglandins, potassium voltage-gated channels and G protein-coupled receptors (Supplementary Table S12). Moreover, 385 genes including *KLHL35, KIF14, KIF26A, NEURL1*, *MTCL1, SEMA4B,* and *RASIP1* were hypermethylated at ≥ 2 CpG sites (Supplementary Table S12). In addition to the gene body regions, 95.4% of DMCGs were mapped to CpG islands and 92.4% of DMCGs observed in different classes of repeats were hypermethylated in MET_noP compared to CON_noP cows (Figures 9C, D). Thus, considering those cows that did not get pregnant following artificial insemination, RPM supplementation caused increased methylation levels on the exonic and intronic regions, CpG islands as well as different classes of repeats.

### 3.11 Effects of RPM supplementation on the endometrial DNA methylation patterns irrespective of pregnancy outcomes

**FIGURE 10:**
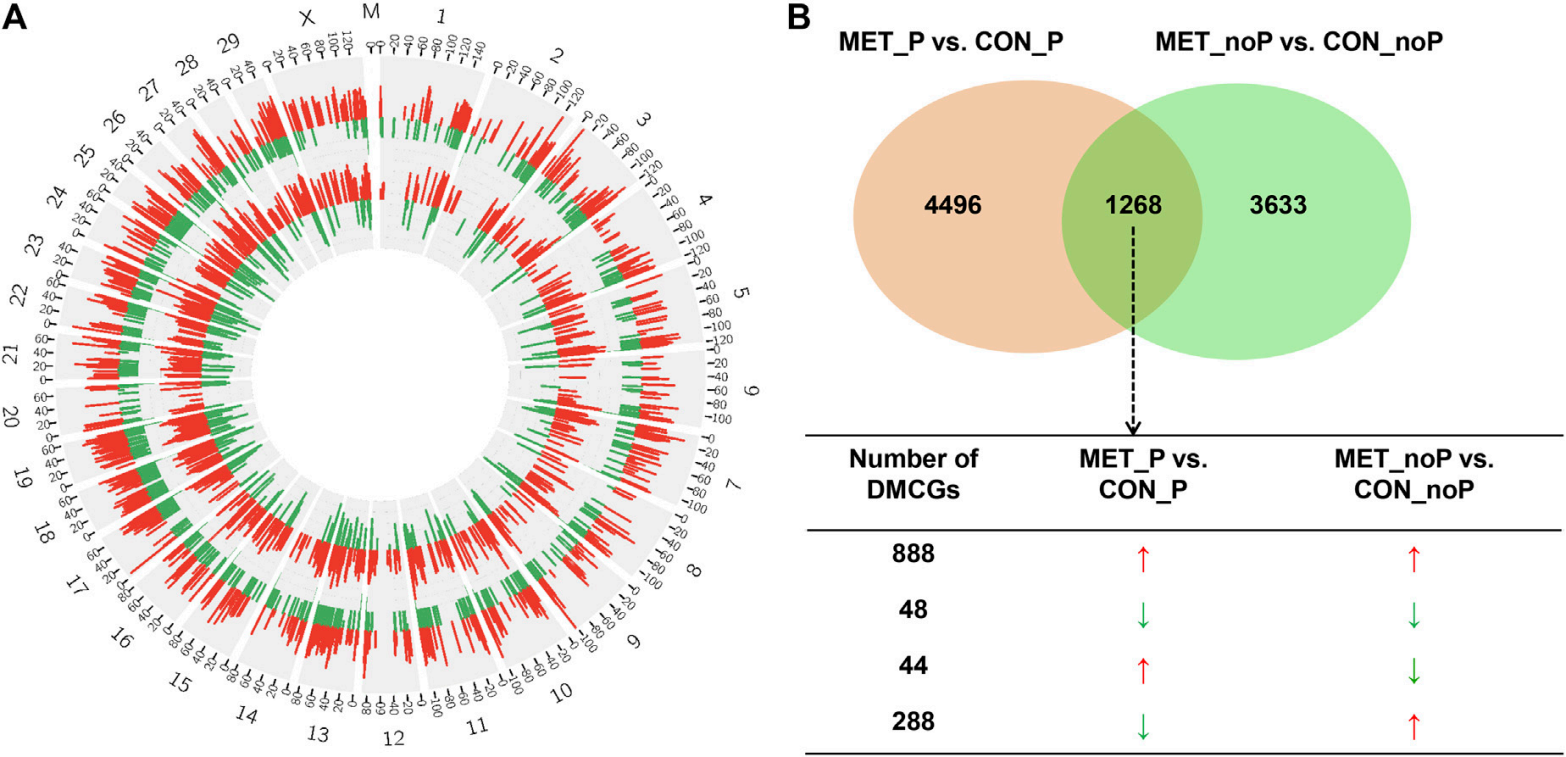
Exclusively and commonly differentially methylated CpG sites in MET_P vs. CON_P and MET_noP vs. CON_noP. **(A)** Circos plot showing chromosome-wide endometrial DNA methylation differences between MET_P and CON_P (outer circos) and between MET_noP and CON_noP (inner circos). Red and green bars represent the number of hypermethylated and hypomethylated DMCGs, respectively. Numbers 1–29, X and M indicate autosomal, X and mitochondrial chromosomes, respectively. **(B)** Commonly and exclusively differentially methylated CpG sites in MET_P vs. CON_P and MET_noP vs. CON_noP. Arrows: ↑ and ↓ on indicate the hypermethylation and hypomethylation, respectively. Circos plots were generated using the circos software (http://circos.ca/software/download/circos/), licensed under GNU General Public License (GPL) v3 (Krzywinski, M. et al. 2009).

To identify the effect of daily RPM supplementation on endometrial epigenetic profiles in relation to pregnancy outcome, we identified commonly and exclusively differentially methylated CpG sites in MET_P vs. CON_P and MET_noP vs. CON_noP cows. Chromosome-wide DNA methylation profiles plotted in 20 Mbp windows revealed the presence of common DMCGs in both comparisons (Figure 10A). On a numerical basis, 1,268 DMCGs were commonly detected in MET_P vs. CON_P and MET_noP vs. CON_noP comparisons (Figure 10B) indicating that these DMCGs were affected by daily RPM supplementation independent of the cow’s fertility status. Interestingly, 888 and 48 DMCGs were hypermethylated and hypomethylated, respectively in MET_P compared to CON_P cows as well as in MET_noP compared to CON_noP cows. Conversely, 79% of DMCGs identified in MET_P compared to CON_P cows were not differentially methylated in MET_noP compared to CON_noP cows. Likewise, 74% of DMCGs identified in MET_noP compared to CON_noP cows were not detected in MET_P vs. CON_P cows (Figure 10B) suggesting the DMCGs were affected by confounding factors of RPM supplementation and the fertility status of cows.

A deeper analysis of these DMCGs with respect to their overlapping genes indicated 453 annotated genes to be differentially methylated between the MET_P and CON_P groups as well as between the MET_noP and CON_noP groups. Among these, 198 genes including *ZNF787*, *ZNF75D*, *ZNF274*, *SOX9*, *PXDN*, *PTGIS, ATP8B1,* and *AGO2* showed increased methylation in the RPM-supplemented group compared to the control diet group (Supplementary Table S13). Functional enrichment analysis revealed that these genes were involved in five pathways including axon guidance, notch signalling and collagen formation (Supplementary Figure S5). On the other hand, 867 genes including those involved in metabolic pathways, focal adhesion, signal transduction, glycerophospholipid metabolism, Wnt signalling and tight junction were differentially methylated exclusively between MET_P and CON_P cows, but not between MET_noP vs. CON_noP cows. Likewise, a total of 610 genes including those involved in the Rap1 signalling pathway, regulation of actin cytoskeleton, phospholipase D signalling pathway and endocrine resistance were found to be differential methylated exclusively between MET_P and CON_P but not between MET_noP and CON_noP group (Supplementary Figure S6).

Once we recognized the presence of endometrial methylation signatures which could be correlated with subsequent pregnancy outcomes at the time of breeding in the presence and absence of methionine feeding, we wanted to know if methionine feeding can the epigenome profile into the directions of higher fertility. To identify differentially methylated genes due to methionine feeding and positively correlated with the fertility status of dairy cows, we, therefore, looked into commonly differentially methylated genes in relation to the pregnancy outcome and methionine feeding. Interestingly, our analysis revealed 19 genes including *ZNF75D*, *RGS3a,* and *AGO2* were differentially methylated due to methionine feeding and pregnancy establishment (Table 5).

**TABLE 5:** Differentially methylated genes with respect to fertility and methionine supplementation.

Gene symbol	Gene description	CON_P vs. CON_noP	MET_P vs. MET_noP	MET_P vs. CON_P	MET_noP vs. CON_noP
*TBL1X*	Transducin beta like 1 X-linked	↓	↓	↓	↓
*COL4A2*	Collagen type IV alpha 2 chain	↓	↓	↑	↑
*STARD10*	StAR related lipid transfer domain containing 10	↓	↓	↑	↑
*ZNF75D*	Zinc finger protein 75D	↑	↑	↑	↑
*RF00002*	No description	↑	↑	↑	↑
*CDK5RAP2*	CDK5 regulatory subunit associated protein 2	↑	↑	↓	↓
*SEPT9*	Septin 9	↑	↑	↑	↑
*AGO2*	Argonaute 2, RISC catalytic component	↑	↑	↑	↑
*CACNA1H*	Calcium voltage-gated channel subunit alpha1 H	↑	↑	↓	↓
*RGS3*	Regulator of G protein signaling 3	↑	↑	↓	↓
*LY86*	Lymphocyte antigen 86	↑	↑	↑	↑
*ZMIZ1*	Zinc finger MIZ-type containing 1	↑	↑	↑	↑
*COL5A1*	Collagen type V alpha 1 chain	↑	↑	↓	↓
*KAT2B*	Lysine acetyltransferase 2B	↑	↑	↑	↑
*NFIX*	Nuclear factor I X	↑	↑	↑	↑
*CCDC88C*	Coiled-coil domain containing 88C	↑	↑	↓	↓
*CUX1*	Cut like homeobox 1	↑	↑	↓	↓
*CAMK2B*	Calcium/calmodulin dependent protein kinase II beta	↑	↑	↑	↑
*AFF3*	AF4/FMR2 family member 3	↑	↑	↓	↓

*Arrows,* ↑ *and ↓ indicate hypermethylation and hypomethylation in the former compared to the latter group*.

## 4 Discussion

Although milk production has increased in modern dairy cows, maintaining their fertility has been a greater challenge. In fact, within the same breed, some of the cows are tolerant and can exit from their negative energy balance in a short time while others may stay longer at the NEB status. These phenomena negatively affect the response of oestrous synchronization, breeding and subsequent pregnancy success. It has been frequently marked that during the early lactation period, elevated concentrations of beta hydroxybutyrate (ßHB) and non-esterified fatty acids (*NEFA*) during NEB (Butler and Smith, 1989) affects the fertility of the postpartum cow by altering the gene expression patterns in the maternal environment, such as the endometrium (Wathes et al., 2007; Fenwick et al., 2008; Wathes et al., 2009). Identification of endometrial molecular indicators of physiological changes due to external and internal conditions (e.g., NEB) may enlighten our understanding of molecular mechanisms of pregnancy establishment. Previously, possibilities of identifying endometrial mRNA and microRNA expression patterns that are correlated to the pregnancy outcome by taking advantage of endometrial samples collected before embryo transfer have been outlined (Salilew-Wondim et al., 2010; Ponsuksili et al., 2012; Ponsuksili et al., 2014). Although prediction of a cow’s fertility based on molecular signatures remains challenging, here we studied the global and site-specific endometrial DNA methylation patterns in postpartum dairy cows in relation to the pregnancy outcome in the presence or absence of RPM using a reduced representation bisulfite sequencing approach.

### 4.1 Endometrial DNA methylome signatures as indictors for fertility in postpartum dairy cows offered a control diet

Identification of endometrial molecular markers such as epigenetic signatures established in the endometrium in the expectation of an incoming embryo in the uterus helps to plan a road map towards the identification of endometrial DNA methylation marks associated with cattle fertility. In the current study, in the control diet group, endometrial DNA methylation analysis at the time of breeding indicated differential methylation of more than 1900 CpG sites between cows that resulted in pregnancy (CON_P) and those that resulted in no pregnancy (CON_noP) following artificial insemination. Interestingly, 45.5% of differentially methylated CpG sites (*n* = 892) were located on chr27: 6,217.2–6,225.6 kb and hypermethylated in the CON_P compared to CON_noP (Supplementary Table S14). This may suggest that the methylation level of this genomic region might be associated with postpartum dairy cow fertility at the time of breeding. These results are in line with our previous studies which demonstrated the pretransfer endometrial transcriptome profile difference between receptive and non-receptive heifers (Salilew-Wondim et al., 2010; Ponsuksili et al., 2012; Ponsuksili et al., 2014). Furthermore, increased methylation levels in about 90% of the DMCGs identified in CON_P vs. CON_noP in the former group may suggest the presence of endometrial epigenetic remodelling differences which could be directly or indirectly related to postpartum cow’s fertility. In this regard, previous studies also reported changes in the endometrial DNA methylation patterns between proliferative and secretory phases in humans (Houshdaran et al., 2014) and the association between uterine DNA methylation alteration and reproductive failures in mice (Woods et al., 2020).

Interestingly, 27.5% of differentially methylated CpG sites were localized in gene body regions (exons and introns) with the majority of them found to be hypermethylated in cows that became pregnant following artificial insemination. Although the implications of this finding for postpartum cow’s fertility status during the breeding time remain vague, increased methylation levels within given gene body regions could be associated with triggering transcription elongation (Jones, 2012). Furthermore, 313 genes that were associated with DMCGs were identified and the majority of them showed higher methylation levels in cows that resulted in pregnancy (Figure 3; Supplementary Table S2). Thus, tracking the methylation record of these genes could also provide insight into using differentially methylated genes and differentially methylated CpG sites for developing DNA methylome markers for fertility. For instance, *LMTK3*, *PRDM16*, and *TMEM170A* were among the top genes exhibiting a significant increase and *RBM19*, *TPST2,* and *SYT2* showed a significant decrease in methylation level in cows resulting in pregnancy compared to those resulted in no pregnancy. Previous studies have indicated the importance of *RBM19* for preimplantation embryo development (Zhang et al., 2008) and *TPST2* for male fertility (Borghei et al., 2006). Although the direct relevance of these genes to the postpartum cow fertility requires further studies, the findings of the present study as well as others indicated that differential methylation of these genomic loci may be related to the fertility of postpartum cows.

In addition to specific differentially methylated CpG sites or genes, we also identified specific genomic regions on chromosomes 27, 18, and 21 that showed hypermethylation patterns in several CpG sites in cows that resulted in pregnancy compared to those that resulted in no pregnancy. For instance, a total of 892 of 1,057 CpG sites (84.3%) located on chromosome 27: 6,217.2–6,225.6 kb, and 108 out of 124 CpG sites (87%) located on chromosome 18:59225.2–59226.0 kb and 58 out of 258 CpG sites (23.4%) located on chromosome 21:33002.6–33028.9 kb were hypermethylated in cows that resulted in pregnancy compared to those resulted in no pregnancy (Supplementary Table S14). Notably, these genomic regions are dominated by CpG islands, repetitive elements (simple repeats, low complexity, rRNA, DNA) and transcription start sites (TSS) underlining that these genomic regions might be potential methylome markers associated with postpartum cow fertility.

### 4.2 Endometrial DNA methylation marks associated with pregnancy establishment in cows that received RPM supplementation

Besides investigating the endometrial DNA methylome profiles of postpartum cows at the time of breeding in relation to the pregnancy outcomes in the control diet group, we also performed the same study in postpartum cows supplemented with RPM. It is well known that apart from improving lactation performances, the relevance of RPM supplementation on the fertility of lactating dairy cows has been a special concern, particularly during periods of negative energy balance. Indeed, methionine is responsible for the generation of a key cofactor that is essential for the establishment of DNA-methylations. Maternal supplementation of RPM before conception and/or during the preimplantation period might alter the expression of genes associated with uterine immune responses and metabolism (Guadagnin et al., 2021) probably via its epigenetic modification effects. In the current study, we compared the endometrial DNA methylation patterns of dairy cows daily supplemented with RPM and resulted in pregnancy (MET_P) compared to those that resulted in no pregnancy (MET_noP). The number of DMCGs identified in MET_P vs. MET_noP was 1.8 times higher compared to those DMCGs identified between cows that resulted in the pregnancy and resulted in no pregnancy in the control diet group (Figure 6). This observation may suggest that supplementation of RPM could enhance epigenome profile differences between postpartum cows that could potentially result in pregnancy compared to those that will fail to be pregnant following artificial insemination during the time of breeding. Although the majority (89%) of DMCGs identified in CON_P vs. CON_noP were hypermethylated and only 11% were hypomethylated (Figure 2), the proportions of hypermethylated and hypomethylated DMCGs in MEP_P vs. MET_noP was nearly equal (48% and 52%, respectively) (Figure 4). But when we look into the numbers of DMCGs, RPM supplementation increased the hypomethylated DMCGs from 217 to 1,777 but decreased the hypermethylated ones from 1741 to 1,653 in cows that resulted in pregnancy compared to those that resulted in no pregnancy (Figures 3, 4). This may in turn suggest the confounding effects of both RPM supplementation and the fertility status of cows during the breeding time. Moreover, from these results, it could be also possible to generalize that methionine supplementation may amplify the endometrial DNA methylation profile differences between cows that could end up in pregnancy and those that resulted in no pregnancy at all. Collectively, this data provides an impression that postpartum cows that could potentially result in pregnancy and no pregnancy can be distinguishable based on their endometrial DNA methylation patterns during the breeding time and supplementation of RPM could further have increased the methylation profile difference between the two cows groups.

### 4.3 Methionine-induced endometrial DNA methylation patterns that are associated with postpartum cow pregnancy outcomes

We believed that differentially methylated CpG sites or genes only between cows that resulted in pregnancy and no pregnancy in the RPM group, not in the control diet may be related to RPM supplementation. In this regard, 2,554 differentially methylated CpG sites between cows that resulted in pregnancy and those that resulted in no pregnancy following RPM supplementation (Figure 6B) were not significantly differentially methylated between cows that resulted in pregnancy and those that resulted in no pregnancy in the control diet group. This may suggest that the methylation patterns of these genomic loci/genes may be highly sensitive to methionine availability. For instance, the methylation patterns of insulin-like growth factor (*IGF1R, IGF2R*), interleukins (*IL17RB, IL17REL, IL34, and IL4I1*) and integrins (*ITGA4, ITGA9, ITGB4, and ITGBL1*) were increased in RPM supplemented cows that resulted in pregnancy compared to those resulting in no pregnancy. Previous studies have shown that *IL17RB* can be associated with endometrial ageing and age-related infertility and *IL34* could be essential fo monocytes/macrophages proliferation or differentiation (Wang et al., 2021a) and induction of decidual macrophages during normal pregnancy (Lindau et al., 2018). Likewise, the expression of endometrial integrins, namely *ITGA9*, *ITGB4,* and *ITGB1* in endometrial epithelial and stoma cells in pig uterus during pregnancy (Zeng et al., 2019) and in the mouse uterus during oestrus (Park et al., 2017) may also indicate the role of these genes in fertility. Furthermore, concerning insulin growth factor receptors, the importance of *IGF1R* for normal epithelium differentiation (Zhou et al., 2021), proliferation and embryo implantation (Sekulovski et al., 2020) have been discussed. On the other hand, our study revealed decreased endometrial methylation levels of CpG sites located on forkhead box *(FOXK2, FOXN1, and FOXN3),* heat shock proteins *(HSPA1A, HSPB7)* and prostaglandin I2 receptor *(PTGIR, PTGER4)* in methionine fed cows that resulted in pregnancy compared to no pregnancy group. The relevance of these genes in fertility has already been documented in many instances although information about their sensitivity to methionine availability is still scarce. Nevertheless, studies by (Gillio-Meina et al., 2009) and others indicated *PTGIR* could be involved in decidualization during the peri-implantation period (Cammas et al., 2006; Blitek et al., 2015), and *FOXN3* may be required for cell proliferation, apoptosis and pathogenesis processes (Sun et al., 2016; He et al., 2019; Wang et al., 2021b). Moreover, lower the expression trend of *HSPA1A* in ectopic compared to eutopic endometrium (Wu et al., 2006) as well as increased expression of *HSPA1A* mRNA in heifers compared to lactating dairy cows (Pretheeban et al., 2011) have also been documented. Therefore, differential methylation of these genes and others between MET_P and MET_noP but not between the CON_P and CON_noP groups may suggest that RPM supplementation can regulate the fertility of the postpartum cow at the time of breeding.by modulating its endometrial epigenome profile.

### 4.4 Endometrial DNA methylation patterns associated with pregnancy establishment irrespective of the feeding regime

To identify DNA methylation marks that can be associated with the fertility status of cows at the time of breeding in those supplemented with RPM or fed a control diet, we analysed commonly differentially methylated CpGs. Notably, the methylation patterns of 414 and 23 CpG sites were increased and decreased in cows that resulted in pregnancy compared to those that resulted in no pregnancy in the control diet and RPM supplemented group. Under our experimental conditions, these results indicated that the endometrial methylation patterns of these CpG sites in postpartum cows at the time of breeding may correlate with a cow’s fertility status but not with the feeding type. In line with this observation, 149 common genes, including *AGO2*, *MP6*, *AFF3*, *CDK5RAP2,* and *DGKD* were differentially methylated between the cows that resulted in pregnancy and no pregnancy independent of whether supplemented with RPM or fed a control diet Previous studies have also indicated differential endometrial expression of *AFF3* between pregnant heifers representing high fertility and non-pregnant heifers (Moraes et al., 2018). Similarly, *BMP6* has been reported to promote female fertility by improving the oocyte quality (Sugiura et al., 2010) or by regulating the expressions of downstream genes in granulosa cells such as the FSH receptor, inhibin/activin beta subunits, and anti-Müllerian hormone (*AMH*) (Shi et al., 2009). Moreover, *BMP6* has been proven to regulate steroidogenesis, peptide secretion and proliferation of antral follicles (Glister et al., 2004) and its dysregulation is associated with pathological endometrium conditions (Athanasios et al., 2012). Similarly, *CDK5RAP2,* a gene that encodes a regulator of CDK5 (cyclin-dependent kinase 5) activity, is important for maintaining the germ cell pool during embryonic development (Zaqout et al., 2017), appropriate chromosome segregation as well as mitotic spindles formation (Zhang et al., 2009). Moreover, DGKD, a gene which encodes an enzyme that phosphorylates diacylglycerol to produce phosphatidic acid, is implicated in central nervous system development and functioning (Leach et al., 2007).

### 4.5 Effects of RPM supplementation on endometrial DNA methylation marks in cows that resulted in pregnancy or resulted in no pregnancy

Once we realized the presence of DNA methylation profile differences between cows that resulted in pregnancy and no pregnancy in the presence or absence of RPM supplementation, we concluded that these two cow groups could be distinguishable based on their endometrial DNA methylation patterns. It was quite clear that RPM supplementation has increased the DNA methylation profile differences between cows that could potentially result in pregnancy and that would result in no pregnancy. We believe that these differences could be induced partly by fertility status and partly due to RPM supplementation. Thus, to know exactly the effect of RPM supplementation on endometrial DNA methylation of postpartum cows during the breeding time in relation to pregnancy establishment, we compared the endometrial DNA methylation profile of cows that become pregnant with RPM supplementation (MET_P) with that of cows that become pregnant without RPM supplementation (CON_P). Likewise, we also compared the endometrial DNA methylation patterns of cows that did not become with RPM supplementation (MET_noP) and those that did not become pregnant without RPM supplementation (CON_noP). With this respect, the results have shown supplementation of RPM may potentially affect the endometrial DNA methylome profile in both cow groups although the effect seems to be higher in cows that resulted in no pregnancy.

Although supplementation of RPM impacted the endometrial DNA methylome during the breeding time in cows that resulted in pregnancy and no pregnancy differently, differential methylation of 453 CpG (Figure 10) sites in both cow groups indicated that the endometrial DNA methylation patterns of these genomic loci in the postpartum cow are mainly affected by RPM supplementation, not by fertility status of cows. Nevertheless, 79% and 74% of the DMCGs identified in MET_P vs. CON_P and MET_noP vs. CON_noP (Figure 10B), respectively were specific to each comparison. The methylation patterns of these genomic loci can be associated with fertility status and RPM supplementation. Indeed, as suggested previously, methionine supplementation may increase the DNA methylation levels in specific genomic regions (Waterland, 2006). In the current study, RPM supplementation was found to induce endometrial methylation levels in cows that resulted in no pregnancy more frequently than in cows that resulted in pregnancy**.** It is believed that aberrant increased DNA methylation could be deleterious (Waterland, 2006). However, further study could be required to investigate whether a relatively higher methylation level induced by RPM supplementation is associated with improvement of the endometrial ecosystem or not.

Finally, in the current study, 19 genes including ZNF75D, RGS3, and AGO2 (Table 5) were commonly differentially methylated due to methionine feeding and pregnancy establishment. These genes could be correlated with subsequent pregnancy outcomes and methionine feeding. Various previous studies have shown the implication of these genes in various cellular processes including follicular and embryonic development. For instance, *ZNF75D* is believed to regulate the activity of androgen receptors, *Smad3*/4, and *p53* (Castillo-Castellanos et al., 2021) and this gene is essential for embryonic viability and proper vascular development (Beliakoff et al., 2008). Similarly, the regulator of G protein signalling 3 (RGS3) was reported to be differentially expressed between oocytes of follicles leading to pregnancy and developmental failure in humans (Hamel et al., 2010). More interestingly, Argonaute 2 (AGO2), a gene that interacts with the dicer RNA interference pathway and miRNA biogenesis, is implicated in early murine development (Morita et al., 2007).

## 5 Conclusion

In this study, we have characterized and identified endometrial DNA methylation landscapes in relation to the pregnancy outcomes in postpartum cows supplemented with RPM or fed a control diet. The results from the present study highlighted that either in the presence or absence of RPM supplementation, the postpartum cows that can be pregnant can be distinguished from those that resulted in no pregnancy based on their endometrial epigenome signatures at the time of breeding. These differences may indicate the presence of genome remodelling in the endometrial environment in expectation of the growing embryo. Furthermore, RPM supplementation has shown incremental effects on the DNA methylation patterns of cows that resulted in no pregnancy than those that became pregnant although supplementation of methionine altered the endometrial epigenome profile of both cow groups. In addition, a set of 19 differentially methylated genes were identified in all experiments and these genes might be fundamentally correlated with cow fertility status as well as methionine supplementation. These genes might be attractive candidates for further studies related to nutrigenomics for improving dairy cows.

Overall, the current study highlighted that postpartum cows that could potentially become pregnant and those that could potentially fail to be pregnant are distinguishable based on their endometrial DNA methylation patterns at the time of breeding. Eventually, these results may provide an opportunity to further navigate and screen promising endometrial DNA methylome markers which may be strongly associated with postpartum cow fertility at the time of breeding.

## Data Availability

The raw fastq data of 48 samples and the semi processed data are stored in the public repository with GEO accession number GSE248185 and bioproject accession number PRJNA1034815.

## Supplementary material

SUPPLEMENTARY FIGURE S1 The global endometrial DNA methylation patterns in cows that result in pregnancy and no pregnancy with and without RPM methionine supplementation when probes are stratified by methylation level of 10% interval.

SUPPLEMENTARY FIGURE S2 The methylation patterns of 890 differentially methylated CpG sites in CON_P and CON_noP sample groups. These CpG sites are located on chr27: 6217.2–6225.6 kb. The bars indicate methylation levels. CON_P_1, CON_P_2, CON_P_3, and CON_P_4 represent four biological replicates from the pregnant group and Av. CON_P indicates the averages from four biological replicates. CON_noP:1, CON_noP_2, CON_noP_3, and CON_noP:4 represent four biological replicates of the non pregnant group and Av. CON_noP indicates the average of the four biological replicates of no pregnant group. RE: Repetitive elements, TSS: Transcriptional start site.

SUPPLEMENTARY FIGURE S3 The chromosomal distribution of 414 hypermethylated and 23 hypomethylated CpG sites in both CON_P and MET_P compared to their counterparts.

SUPPLEMENTARY FIGURE S4 The average methylation patterns of 289 CpG sites located on chr27: 6219560–6225297 in CON_P, CON_noP, MET_P and MET_noP. These CpG sites were differentially methylated between CON_P, and CON_noP, as well as between MET_P and MET_noP.

SUPPLEMENTARY FIGURE S5 Exclusively and commonly differentially methylated genes in MET_P vs. CON_P and MET_noP vs. CON_noP and their functional annotation.
