# Supplementary figures and images for: Endometrial DNA methylation signatures during the time of breeding in relation to the pregnancy outcome in postpartum dairy cows fed a control diet or supplemented with rumen-protected methionine
Source: Front Genet. 2024 Jan 24;14:1267053. doi: 10.3389/fgene.2023.1267053 (PMC10847534; doi:10.3389/fgene.2023.1267053)

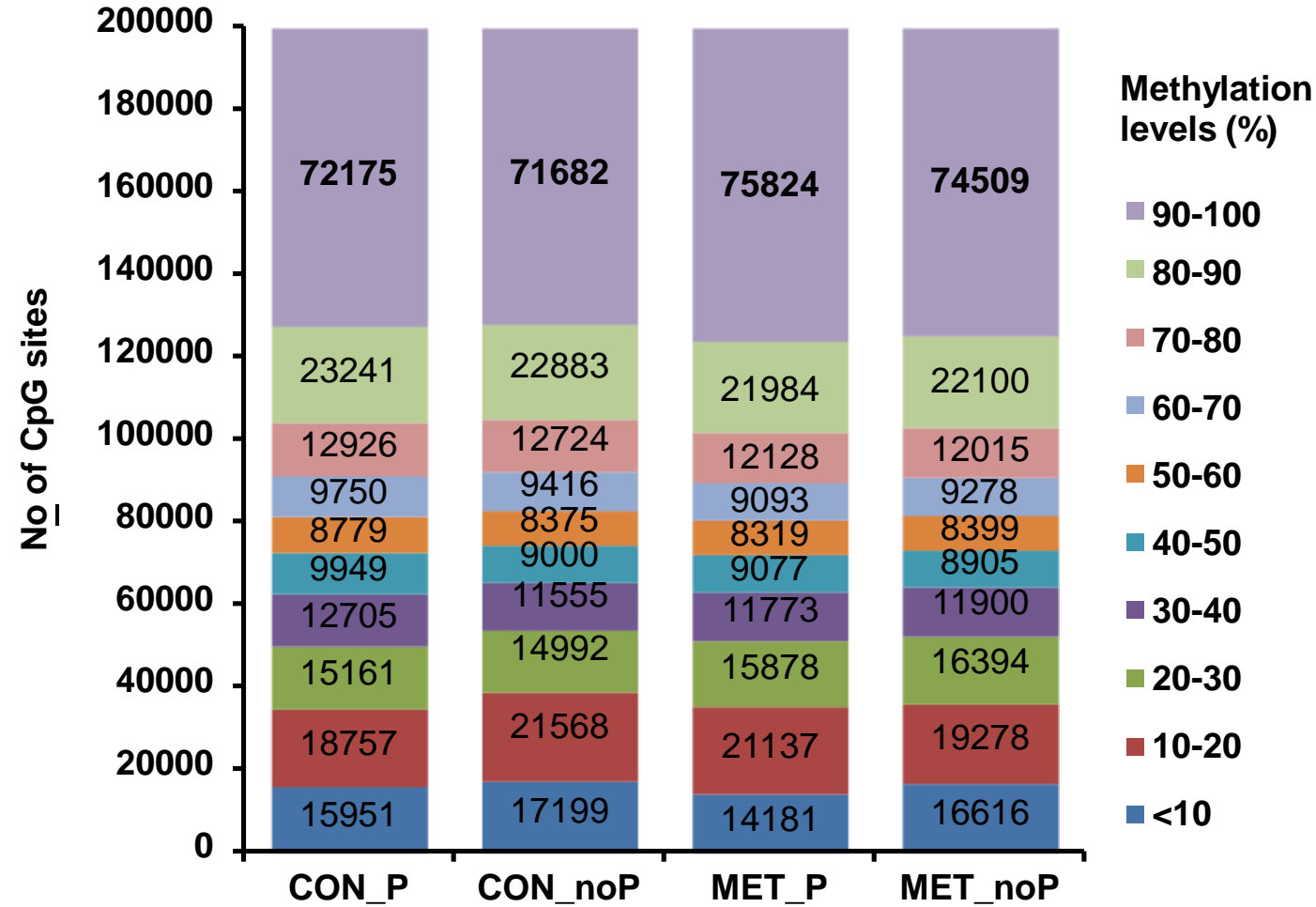

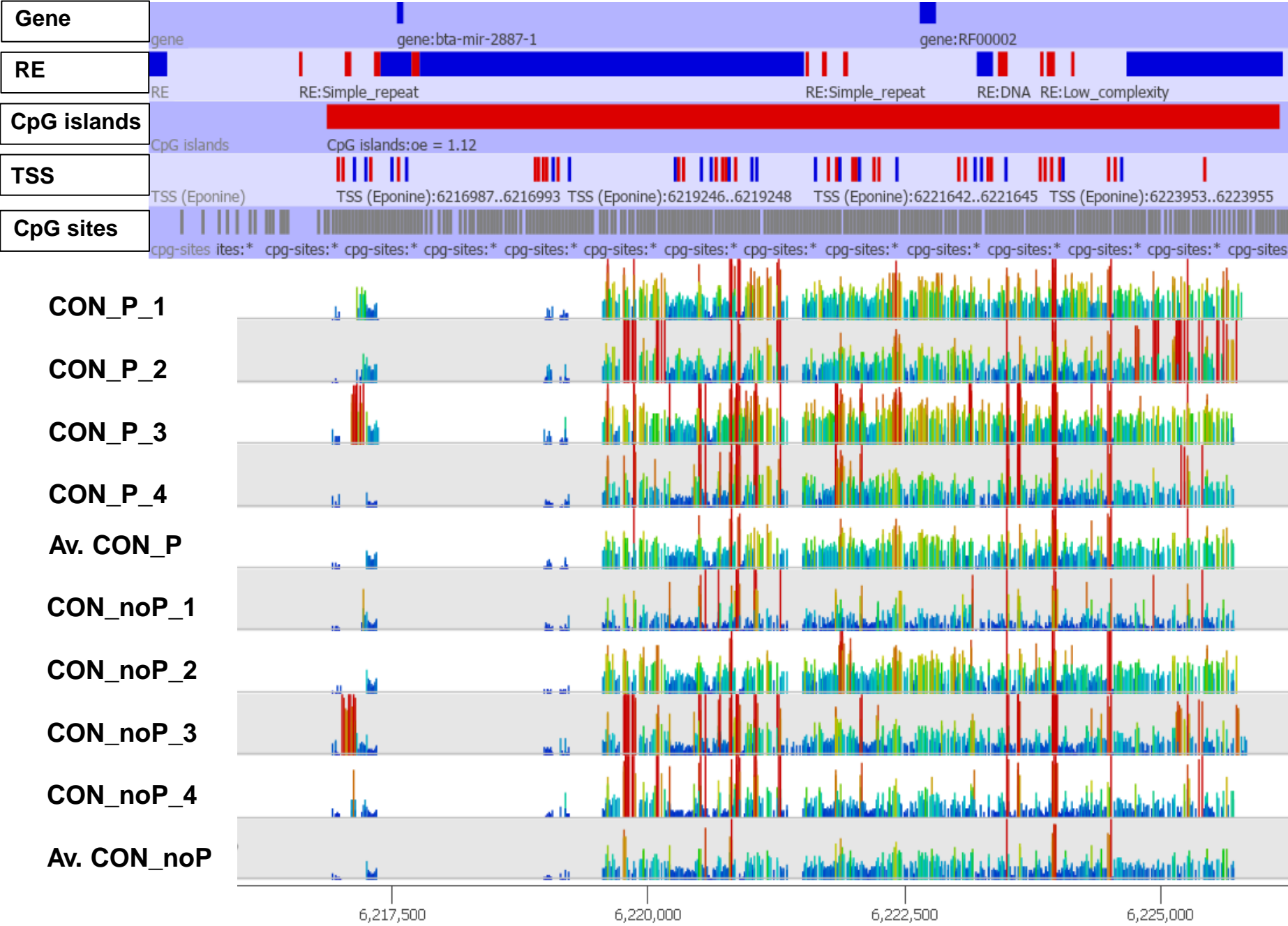

Salilew-Wondim et al., Figure S3

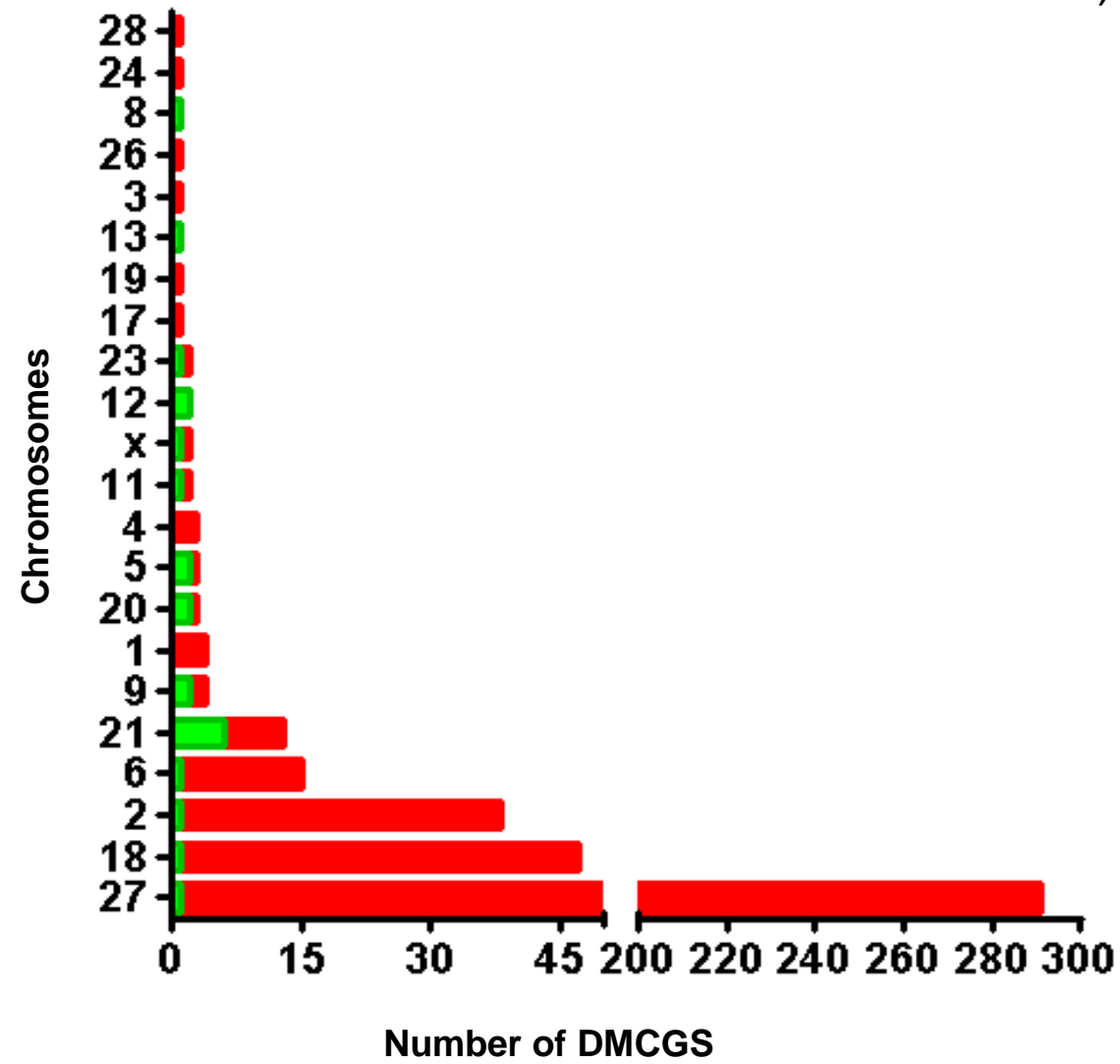

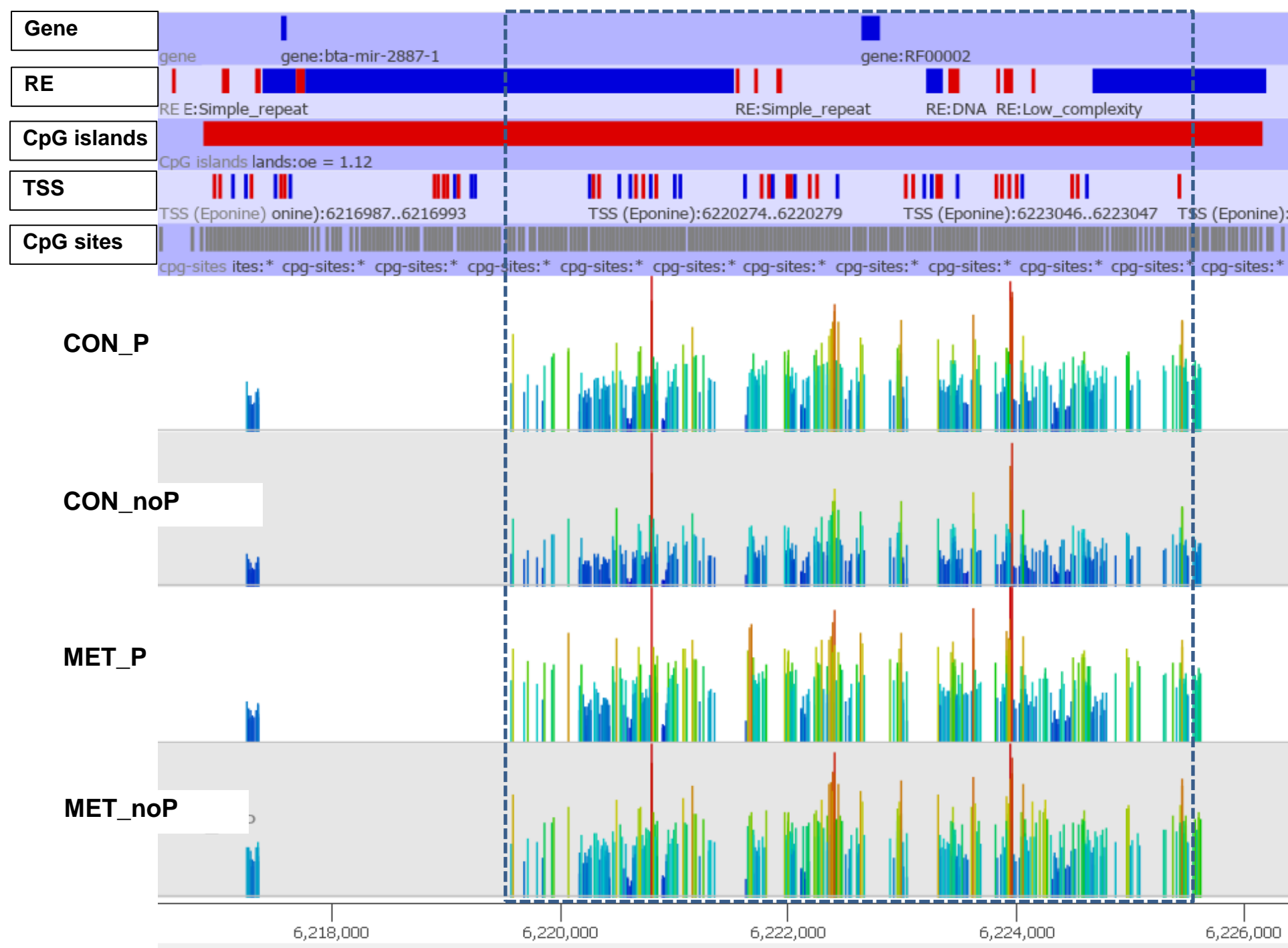

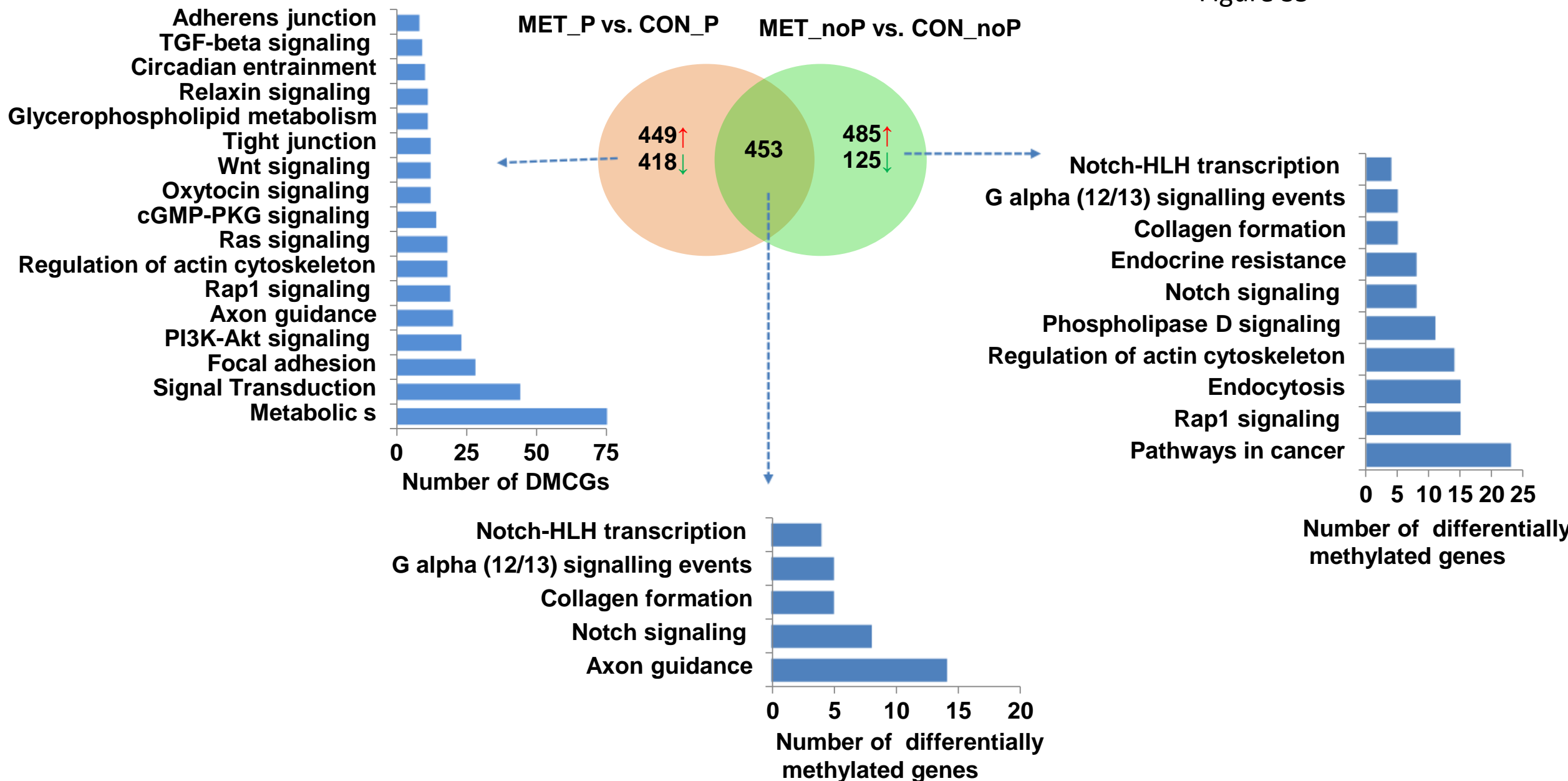

Supplement: Supplementary file 1 [file DataSheet1.PDF]
